# Climatic niche divergence and long-distance dispersal contributed to the pantropical intercontinental disjunctions of a liana lineage (*Uncaria*, Rubiaceae)

**DOI:** 10.1016/j.pld.2025.05.001

**Published:** 2025-05-09

**Authors:** Xian-Han Huang, Jing-Yi Peng, Nan Lin, Jian Liu, Jun-Tong Chen, Qun Liu, Xin-Jian Zhang, Quan-Sheng Fu, Peng-Rui Luo, Zhi-Yu Wang, Shiou Yih Lee, Qiang Zhou, Hang Sun, Tao Deng

**Affiliations:** a Yunnan International Joint Laboratory for Biodiversity of Central Asia, Kunming Institute of Botany, Chinese Academy of Sciences, Kunming 650201, Yunnan, China; b State Key Laboratory of Plant Diversity and Specialty Crops, Kunming Institute of Botany, Chinese Academy of Sciences, Kunming 650201, Yunnan, China; c College of Biology and Environmental Sciences, Jishou University, Jishou 416000, Hunan, China; d College of Life Science, Henan Agricultural University, Zhengzhou 450002, Henan, China; e Faculty of Health and Life Sciences, INTI International University, Nilai 71800, Negeri Sembilan, Malaysia

**Keywords:** Niche evolution, Long-distance dispersal, Pantropical intercontinental disjunction, Miocene, Woody lianas, *Uncaria*

## Abstract

The formation of pantropical intercontinental disjunction (PID) in plants has generally been attributed to vicariance, boreotropical migration, and long-distance dispersal. However, this pattern has primarily been examined in herbs, shrubs, and trees, and less commonly studied in interlayer plant taxa. Here we examined evolutionary processes that resulted in the PID of a pantropical woody liana, *Uncaria* (Rubiaceae). We first constructed a comprehensive phylogeny by employing 73 plastid protein-coding sequences from 29 accessions of *Uncaria* (including 16 newly sequenced) from different continents. We then inferred divergence time, history and ecological niche evolution of this genus. Our results showed that *Uncaria* consisted of four well-supported clades that belonged to two geographically distinct lineages: the Asia-Oceania lineage and the Afro-Neotropical lineage. Biogeographic reconstruction showed this genus likely originated in Asia during the early Miocene (*ca*. 19.03 Ma) and the Middle Miocene Climatic Optimum may have triggered the early diversification of *Uncaria*. Due to its recent origin and small seeds with long wings, wind or water-mediated long-distance dispersal may have contributed to the distribution of *Uncaria* in tropical Oceania (via stepping-stone dispersal) and tropical Africa and America (by transoceanic dispersal). Our findings also indicate that diversification of *Uncaria* was primarily driven by ecological niche divergence, particularly climatic factors. Our study emphasizes the dual role of climatic niche divergence and long-distance dispersal in shaping the PID of *Uncaria*, providing references for many other extant lineages with similar distributions.

## Highlights

• *Uncaria* consisted of four clades forming two geographically distinct lineages: Asia-Oceania and Afro-Neotropics.
• *Uncaria* likely originated in Asia during the early Miocene, making a case for a young origin of liana in pantropical forests.
• Diversification of *Uncaria* was associated with the Middle Miocene Climatic Optimum and climatic niche divergence.
• Wind or water-mediated long-distance dispersal likely contributed to the pantropical distribution of *Uncaria*.

## 1 Introduction

Pantropical intercontinental disjunction (PID), which refers to the connection between tropical areas in Africa, the Americas, Asia, and Australia (Thorne, 1972), has been observed in many plants (Thorne, 1972; Wei et al., 2015; Scheben et al., 2016) and animals (Ye et al., 2017; Selvatti et al., 2022). PIDs have been documented in about 59 families and 334 genera of seed plants (Thorne, 1972). PIDs are thought to have been formed by three main processes: vicariance (often linked to tectonic movements), boreotropical migration (always involved the Paleocene-Eocene Thermal Maximum), and long-distance dispersal (related to the dispersal capability of a particular taxon) (Givnish and Renner, 2004; Wei et al., 2015; Selvatti et al., 2022). In turn, these three processes have been shaped by plate tectonics and climatic oscillations in different geological periods (Viruel et al., 2016). Identifying the origin and diversification of lineages with a PID is key to understanding the evolutionary assembly of pantropical forests (Nie et al., 2013).

The processes that shape tropical disjunctions, including PIDs, remain largely undocumented, especially compared to those that form temperate disjunctions (Manos and Donoghue, 2001; Wen and Ickert-Bond, 2009; Wen et al., 2016). Furthermore, biogeographical studies that have examined taxa with PID patterns have focused on family level disjunctions, e.g., Podocarpaceae (Klaus and Matzke, 2020) and Zamiaceae (Coiro et al., 2023) in gymnosperms, the Myristicaceae (Frost et al., 2022) in magnoliids, the Burmanniaceae (Merckx et al., 2008), Corsiaceae (Mennes et al., 2015), Costaceae (Specht, 2006), and Marantaceae (Prince and Kress, 2006) in monocots, and the Cunoniaceae (Pillon et al., 2021), Loranthaceae (Liu et al., 2018), Melastomataceae (Morley and Dick, 2003), Simaroubaceae (Clayton et al., 2009), and Urticaceae (Wu et al., 2018; Huang et al., 2019) in eudicots. Many of these studies have only examined herbs, shrubs, and/or trees, but neglected one significant physiognomic and structural component of modern tropical rainforests that may offer significant opportunities for investigating rainforest assemblage and evolution, namely, lianas (Gentry, 1991; Schnitzer and Bongers, 2002; Wang et al., 2012). Biogeographic studies of lianas are still relatively scarce, with a couple of cases focused on *Cissus* (Vitaceae; Liu et al., 2013), Cucurbitaceae (Schaefer et al., 2009), Menispermaceae (Wang et al., 2012), and *Paederia* (Rubiaceae; Nie et al., 2013).

**Fig. 1:**
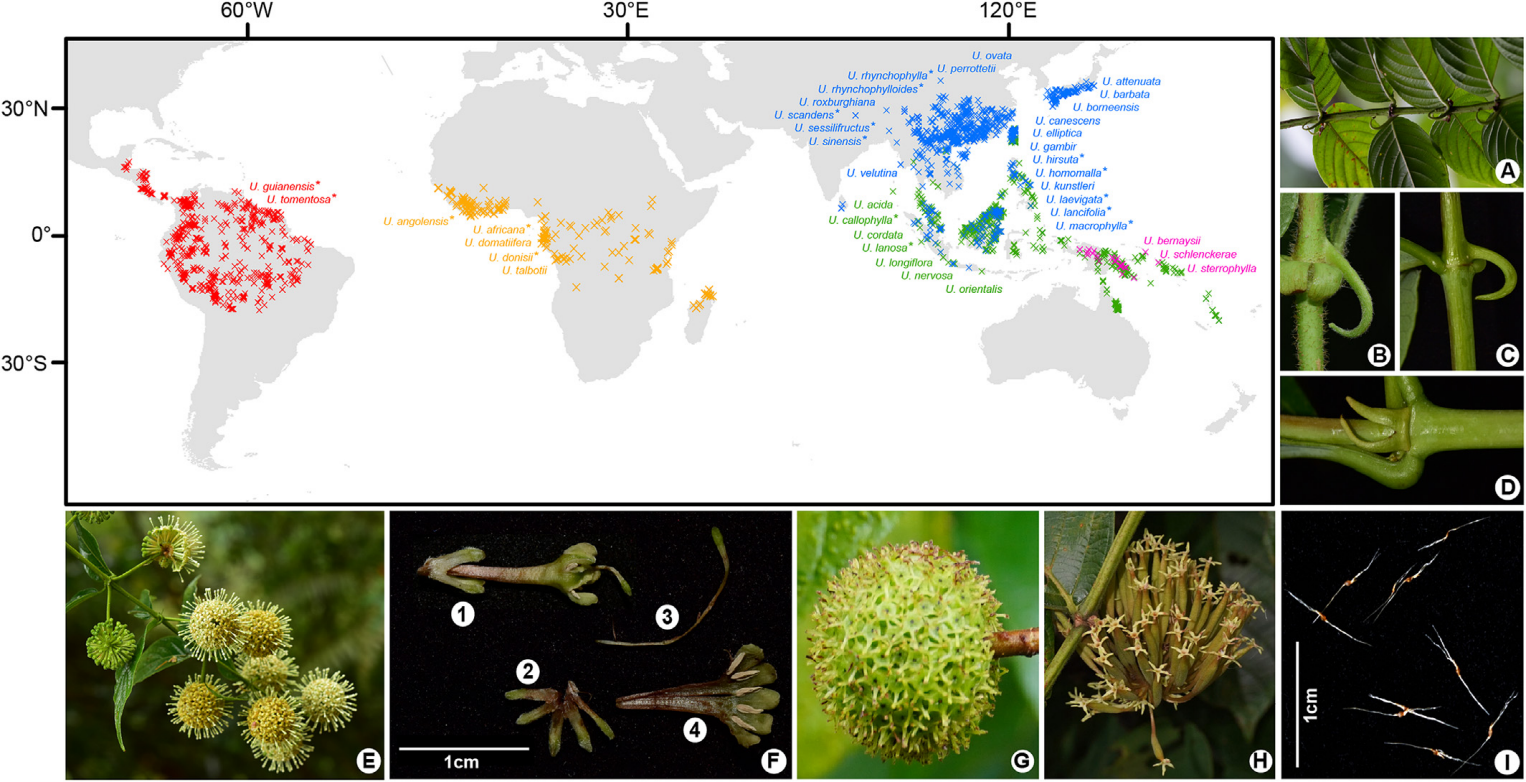
Distribution and morphology of *Uncaria*. Distributional data of 38 *Uncaria* species except *U. ovata* were collected from the Global Biodiversity Information Facility (GBIF, http://www.gbif.org/), the Chinese Virtual Herbarium (CVH, http://www.cvh.ac.cn/), and the sampling sites in this study. The red, yellow, pink, and blue occurrence points (“ × ”) correspond to the distribution ranges of endemic species in tropical America, Africa, Oceania and Asia, respectively; the green points indicate that the areas of species span across tropical Asia and Oceania. Species with “∗” in their names were sampled in this study. Photos: (A) Hooked stem and opposite leaves of *U. lancifolia*; (B, C) Hairy stem of *U. hirsuta* vs. smooth stem of *U. rhynchophylla*; (D) Bifid stipule of *U. rhynchophylla*; (E) Inflorescence of *U. homomalla*; (F) Flower anatomical diagram of *U. lancifolia*, F1: Sessile flower with calyx, F2: Five stamens in corolla, F3: Style, F4: Calyx; (G) Sessile fruit head of *U. hirsuta*; (H) Fruit head of *U. macrophylla*, showing pedicellate fruits; (I) Winged seeds of *U. angolensis*.

An ideal taxon for studying how PID patterns formed is the woody liana genus *Uncaria* Schreb. in the Rubiaceae family, one of the ten most dominant liana families in tropical rainforests (Senbeta et al., 2005; Wang et al., 2012). The genus consists of 38 predominantly pantropical species (Chen and Taylor, 2011; POWO, 2024) that can be easily recognised and distinguished by morphological traits, including recurved spines, corolla tube length, flower and fruit pedicels, interfloral bracteoles, leaf hairs, and shape and division of stipules (Ridsdale, 1978; Chen and Taylor, 2011) (Fig. 1). The diversity centre of *Uncaria* is Asia. Furthermore, seven *Uncaria* species are distributed from Asia to tropical Oceania, five in tropical Africa, three in tropical Oceania, and two in tropical America (Ridsdale, 1978; Chen and Taylor, 2011; POWO, 2024). The processes that drove the current distribution of these species remain unclear.

Phylogenetic analyses based on morphological and molecular data have recently placed *Uncaria* into the tribe Naucleeae (Razafimandimbison and Bremer, 2001). Unfortunately, infrageneric relationships derived from previous studies based on nuclear and plastid markers have been shown to be poorly supported, and the relationships between most species remain ambiguous (Zhang et al., 2015; Zhu et al., 2018; Liu et al., 2023). More recent phylogenomic studies based on plastid genomes (plastomes) have elucidated the interspecific relationships of a couple of Chinese *Uncaria* (species recorded in China), and American indigenous species (*U. tomentosa* (Willd. ex Schult.) DC., and *U. guianensis* (Aubl.)) (Chen et al., 2022; Castro et al., 2023; Dai et al., 2023). However, understanding the biogeographical history of *Uncaria* requires additional phylogenetic analysis with increased taxon sampling, especially from different continents.

In this study, we aimed to identify the evolutionary processes that formed a pantropical intercontinental disjunction in the liana genus *Uncaria*. For this purpose, we used plastomes and extensive sampling from multiple continents to generate a comprehensive phylogeny of *Uncaria*. We combined our phylogenetic analysis with biogeographical reconstruction, and diversification dynamics to elucidate the spatiotemporal diversification dynamics of this genus at the global scale. Lastly, we integrated ecological niche evolution and geological history of the genus.

## 2 Materials and methods

### 2.1 Taxon sampling and morphological character collection

A total of 20 plastomes were newly sequenced and assembled, including 16 samples from 13 *Uncaria* species and four from related genera, *Neolamarckia cadamba* (Roxb.) Bosser, *Nauclea orientalis* (L.) L., *Nauclea latifolia* Sm., and *Cephalanthus occidentalis* L. (Table S1). In this study, our sampling combined with 13 publicly available *Uncaria* plastomes (Table S2) reached a species-level coverage of 45% (17/38 species; POWO, 2024), including two infraspecific taxa of *U. lanosa* Wall. Our dataset encompasses *Uncaria* species from all continents with occurrence records, including both endemic species from tropical America, three of five endemic species from tropical Africa, half the endemic species from Asia (including *U. lanosa* f. *philippinensis* (Elmer) Ridsdale, 11/21), and two of seven species that are distributed widely from Asia to tropical Oceania (including *U. lanosa* var. *appendiculata* (Benth.) Ridsdale). Despite extensive efforts, three Oceanian endemics, namely *U. bernaysii* F. Muell., *U. sterrophylla* Merr. & L.M. Perry, and *U. schlenckerae* S. Moore, confined to New Guinea and its surrounding islands, remain unsampled. Collection details, voucher information, and GenBank accession number are provided in Table S1.

Based on the Flora of China (FOC; Chen and Taylor, 2011) and revision of *Uncaria* by Ridsdale (1978), seven key morphological characters were compiled for all recognised species within the genus (Table S3). Overall, the 17 sampled species adequately represent the morphological variation of the following traits: presence or absence of hairs on the corolla lobes and corolla tube; corolla tube length (shorter or longer than 5 mm); fruit pedicels (pedicellate or sessile); presence or absence of interfloral bracteoles; presence or absence of leaf hairs; and stipule shape (entire or bifid) (Table S3). These morphological data were derived from the FOC, published literature (Ridsdale, 1978), type specimens, and the Chinese Virtual Herbarium (CVH, https://www.cvh.ac.cn/), as well as field observations and measurements. All morphological characters were coded as binary. For continuous characters, specimens of species lacking recorded data were measured multiple times using ImageJ v.1.54, with priority given to type specimens and voucher numbers documented to ensure data reliability. Based on the classification of *Uncaria* (Ridsdale, 1978), the values used to distinguish between species were set as limits for recording the related features.

### 2.2 DNA extraction, sequencing, assembly and annotation

Fresh leaves were collected during field investigations and dried in silica. Total DNA was extracted from silica-dried leaf at Novogene (Beijing, China). Sequencing libraries were generated using the NEB Next® Ultra DNA Library Prep Kit for Illumina® (Ipswich, Massachusetts, USA), following the manufacturer’s instructions. Prepared libraries were sequenced on the Illumina Hiseq 4000 platform. Plastomes were assembled from clean data in GetOrganelle v.1.7.4.1 (Jin et al., 2020) using the following parameters: –F plant_cp –R 15 -t 8 -k 75, 105, 115, 127. The preliminary scaffolds were visualised in Bandage v.8.1 to assess the quality of the assemblies (Wick et al., 2015). All circular plastome sequences were annotated on the web page GeSeq (Tillich et al., 2017). Annotation results were manually checked and adjusted in Geneious v.9.0.2 (Kearse et al., 2012) with *Uncaria rhynchophylla* (Miq.) Miq. (GenBank: MT991006) as the reference to correct annotation accuracy. Additionally, two plastome sequences, *Uncaria guianensis* (GenBank: OP794339) and *U*. *tomentosa* (GenBank: OP794340), were rectified and re-annotated in this study due to assembly errors with sequence duplication. Complete plastome physical maps were finally drawn in OGDRAW (Greiner et al., 2019).

### 2.3 Phylogenetic analysis and comparative plastid genomics

The plastid phylogenetic matrix comprised 46 sample sequences, with 26 samples (including eight species as outgroups) obtained from GenBank (Table S2). From each sample, 73 shared plastid protein-coding sequences (CDS) were extracted for subsequent phylogenetic analysis, and sequence alignment was conducted using MAFFT (Katoh and Standley, 2013) in Geneious v.9.0.2 with manual adjustments.

Phylogenetic inference was performed using Bayesian inference (BI) and maximum likelihood (ML). For BI analysis, GTR + I+Γ was selected as the best-fitting model based on Akaike information criterion (AIC) in JmodelTest 2 v.2.1.6 (Posada, 2008). The Markov chain Monte Carlo analyses were run with four simultaneous chains of 10,000,000 generations and tree sampling every 1000 generations. After the first 25% of trees were discarded as burn-in, the remaining trees were used to construct a majority-rule consensus tree with Bayesian posterior probabilities by using MrBayes v.3.2 (Ronquist et al., 2012) on the CIPRES Portal (Miller et al., 2010). ML analysis was conducted using IQ-TREE v.2.0.3 with 1000 bootstrap replicates under the optimal model TVM + F + R2, which was selected by Bayesian Information Criteria (BIC) (Nguyen et al., 2015).

Comparative plastid genomics was conducted to investigate potential correlations between plastome evolution and phylogeny of *Uncaria*. We summarised the genome sizes of the whole plastome genome and large single copy (LSC) region for all samples in this study, and simple sequence repeats (SSRs) were identified using the MISA online tool (Beier et al., 2017) to assess structural variation and repeat dynamics across clades. Parameters in MISA were set to ten, five, and four repeats for mononucleotides, dinucleotides, and trinucleotides, respectively. Three repeats were used for tetranucleotide, pentanucleotide, and hexanucleotide.

### 2.4 Divergence time estimation

Divergence-time estimation of *Uncaria* was performed using BEAST v.2.6.7 (Bouckaert et al., 2014) on the CIPRES Portal, with parameters set in BEAUti v.2.6.7. The GTR + I+Γ model was determined as the appropriate substitution model by JmodelTest 2. The uncorrelated lognormal relaxed molecular clock model and the Yule model were selected as clock model and tree prior, respectively, as they are best supported by the nested sampling (NS) method (Russel et al., 2019) implemented in BEAST. Two independent BEAST runs were implemented, each including 200 million generations and sampling one tree from every 1000 generations. The convergence of each run was checked in Tracer v.1.7.2 (Rambaut et al., 2018) to ensure effective sample size (ESS) of all parameters above 200. A burn-in of the initial 25% sampled trees was discarded, and TreeAnnotator was used to produce a maximum clade credibility (MCC) tree.

A review of available fossil records, including those in the Paleobiology Database (https://paleobiodb.org/#/), indicates that no fossils of *Uncaria* have been documented to date. The most reliable fossils from subfamily Dialypetalanthoideae are *Cephalanthus* fruit (Razafimandimbison and Rydin, 2024). These fossils, which have several distinct morphological features, have been reported from ∼20 sites dated between the late Eocene and Pliocene (Friis, 1985; Antonelli et al., 2009). Following Nie et al. (2013) and Yang et al. (2022), the oldest fossil of *Cephalanthus* fruit was used to place a normal constraint on the stem age of this genus with a mean of 33.9 and sigma of 1.0. For our analysis, we used pollen fossils that are consistent with the main fossil evidence of this subfamily, i.e., dispersed pollen grains of the common tricolporate type. Notably, *Scyphiphora* possesses a unique pollen morphology, characterised by distinct pores with a protruding papilla-like rim (Bremer and Eriksson, 2009). Accordingly, the oldest known pollen fossil of *Scyphiphora*, dated to 23 Ma from the Marshall Islands in the northern Pacific Ocean (Leopold, 1969; Saenger, 1998), was used to place a normal constraint on its stem age, with a mean of 23 Ma and sigma of 1.0. To root the tree, the crown age of Dialypetalanthoideae was calibrated using a normal distribution prior with a mean of 54.34 and sigma of 5.0 based on age estimations from Yang et al. (2022) that employed multiple fossil calibrations and incorporated samples from the entire family. To evaluate the robustness of our dating results obtained using normal priors, we conducted additional dating analyses employing both exponential and lognormal priors. In both alternative prior distributions, we consistently set a mean of 1 for two fossil calibrations.

### 2.5 Diversification dynamics analyses

A Bayesian analysis of macroevolutionary mixtures (BAMM v.2.5.0) was constructed to model the speciation and diversification dynamics of *Uncaria* (Rabosky, 2014). To alleviate the effect of incomplete and unequal sampling on diversification rate parameter estimation (Stadler, 2013), species-specific sampling probabilities were applied in each of these three genera as follows: *Uncaria* (0.45, 17 out of the total 38), *Nauclea* (0.17, 2 out of the total 12) and *Neolamarckia* (1.00, 2 out of the total 2). The appropriate priors for the BAMM analysis were estimated using the *setBAMMpriors* function from the package BAMMtools v.2.1.11 (Rabosky et al., 2014) in R v.4.2.3 (Team, 2023). The analysis was run for 50 million generations and sampled at every 10,000. The first 10% of sampled data were discarded as burn-in. The ESSs and convergence were assessed in BAMMtools. Meanwhile, the lineage-through-time (LTT) plots were constructed to visualise temporal dynamics of diversification in *Uncaria* by using the R package APE v.5.7–1 (Paradis and Schliep, 2019). A total of 1000 trees were randomly sampled from the BEAST posterior distribution with outgroups pruned and used to calculate a 95% credibility interval.

### 2.6 Ancestral range reconstruction

To infer the historical biogeography of *Uncaria*, ancestral range estimation was performed using BioGeoBEARS v.1.1.3 (Matzke, 2013a) implemented in R based on the BEAST MCC tree with *Uncaria*, *Nauclea* and *Neolamarckia*. Based on the endemism of *Uncaria* (Ridsdale, 1978; Chen and Taylor, 2011; POWO, 2024) and tectonic histories of continents, we divided the distribution range into four biogeographical regions: A, Asia; B, tropical Oceania; C, tropical Africa; D, tropical America. The number of maximum regions in ancestral ranges was set to two as no extant species occurs in more than two regions. We evaluated two alternative dispersal models: an unconstrained model (M0), in which dispersal probabilities were assumed equal among all areas, and a stratified constrained model (M1; Table S4). Two time slices (20–7 Ma and 7–0 Ma) were defined based on changes in continental connectivity relevant to plant biogeography, particularly the Bering Land Bridge, which facilitated biotic exchanges between 20 and 7 Ma (Tiffney and Manchester, 2001; Viruel et al., 2016). Dispersal probabilities between areas were assigned to three tiers: 1 for dispersal between adjacent areas without barriers (e.g., Asia and tropical Africa), 0.5 for dispersal between areas separated by intermittent barriers (e.g., tropical Africa and tropical Oceania), and 0.01 for highly unlikely dispersal events (e.g., between tropical Oceania and tropical America). For ancestral range reconstruction under the M0 and M1 models, the DIVALIKE model was identified as the best-fitting model (Table S5) among the six evaluated models, including DEC, DIVALIKE, and BAYAREALIKE, along with their “+J” versions (Matzke, 2013b), based on the Corrected Akaike Information Criterion (AICc) values in BioGeoBEARS. In addition, we conducted biogeographical stochastic mapping (BSM) implemented in BioGeoBEARS to estimate the number and type of biogeographical events under the M1 model from 100 BSMs.

### 2.7 Niche modelling and analysis of ecological niche evolution

Except for *Uncaria* *ovata*, occurrence data for the 38 *Uncaria* species were compiled from the Global Biodiversity Information Facility (GBIF, https://www.gbif.org/), our fieldwork records, and specimen information from the Chinese Virtual Herbarium (CVH, http://www.cvh.ac.cn/). Data for each species were manually checked, with duplicates, assumed cultivation records, and other unlikely locations removed. To avoid the effect of uneven distribution data, only one occurrence was kept in each grid cell of 2.5 × 2.5 arc min for 17 sampled *Uncaria* species. A total of 1444 occurrences of the 17 *Uncaria* species were obtained for analysis (Table S6). Environmental factors, including 19 climate variables, five soil variables, and three related variables (wind speed, downward surface shortwave radiation, and vapor pressure), were collected for each species by their location obtained from WorldClim v.2.1 (Fick and Hijmans, 2017), Harmonized World Soils Database v2.0 (Fischer et al., 2008) and Terraclimate (Abatzoglou et al., 2018) at 2.5-arc-minute resolution (Table S6).

We modelled ecological niches for four clades of 17 *Uncaria* species in MaxEnt v.3.4.4 (Phillips and Dudík, 2008) with 10 replicates. The locality data was partitioned into training and testing datasets (75 % and 25%, respectively) to evaluate the quality of the model. For Maxent, the ‘*ENMeval*’ package in R v.4.3 was used to define the optimal model parameters for “regularization multiplier” and “feature class” combinations (Table S7) according to the lowest AICc values. Highly correlated bioclimatic variables were filtered according to the importance of environmental factors from MaxEnt v.3.4.4 (Table S8) with a threshold of 0.3 and Pairwise Pearson’s correlation analysis with a threshold of 0.8 (Fig. S1). Subsequently, a total of eleven factors were retained for niche modelling: (i) mean diurnal range (bio2), (ii) isothermality (bio3), (iii) mean temperature of the wettest quarter (bio8), (iv) annual precipitation (bio12), (v) precipitation of the wettest quarter (bio16), (vi) precipitation of the driest quarter (bio17), (vii) precipitation of the warmest quarter (bio18), (viii) wind speed, (ix) downward surface shortwave radiation, (x) sand content, and (xi) soil texture classification by USDA.

Niche evolution analysis with all 27 factors was used to determine how environmental factors have shaped the evolution of *Uncaria*. The “anc.clim” function of the ‘*phyloclim*’ package in R v.4.3 (Evans et al., 2009) was used to calculate the generalised-least-squares estimate for each environmental variable at each interior node with 1000 random relicates.

## 3 Results

### 3.1 Phylogenetic relationships and comparative plastid genomics

**Fig. 2:**
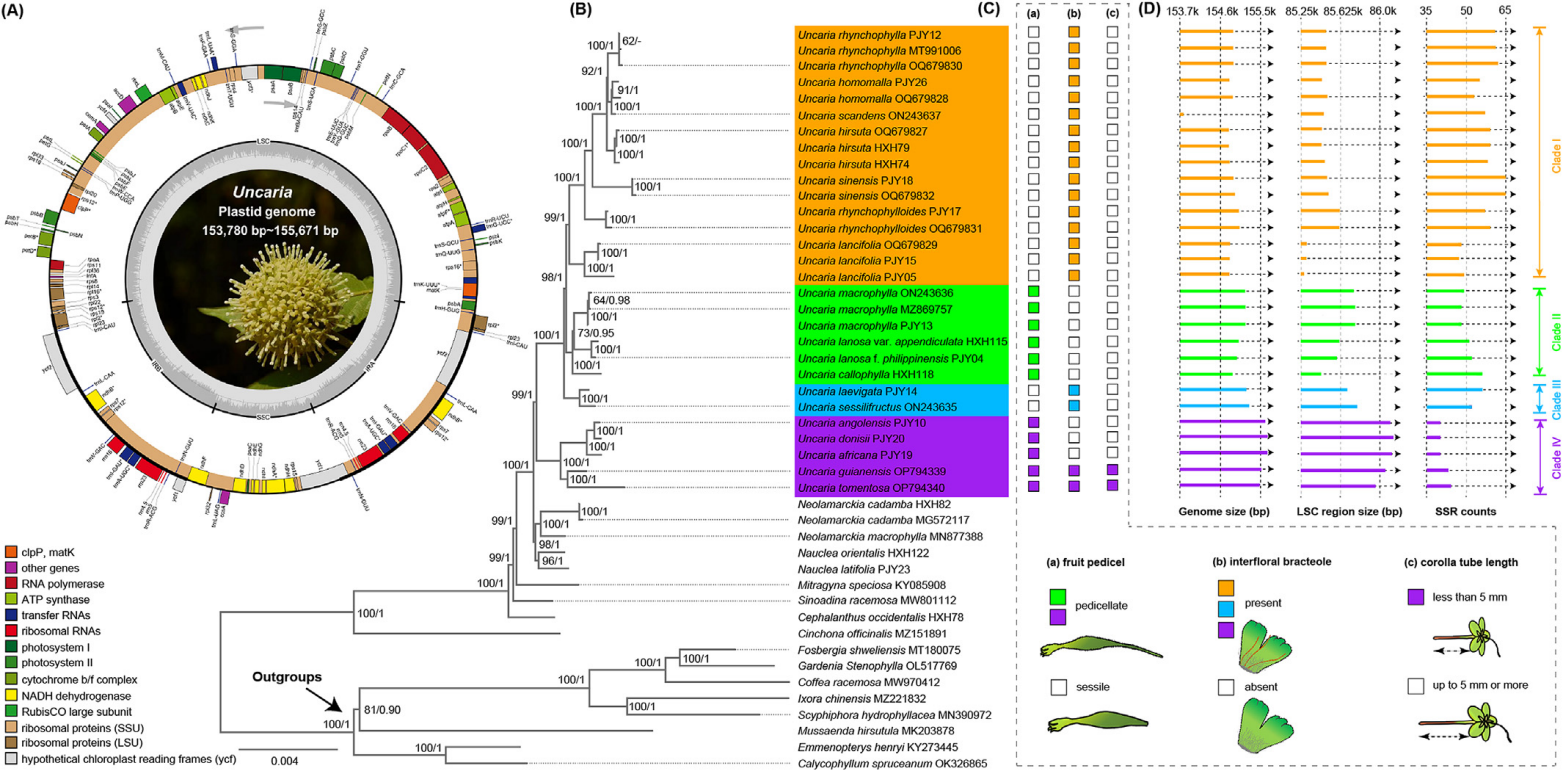
Phylogeny, morphological traits, and plastome features of *Uncaria*. (A) Gene map of *Uncaria* plastid genomes. Genes inside the circle are transcribed clockwise, and those on the outside are transcribed counter-clockwise. Genes belonging to different functional groups have been coded in different colors. The darker grey area in the inner circle corresponds to GC content, and the lighter grey corresponds to AT content. (B) ML phylogenetic tree inferred by 73 shared CDS sequences; numbers at the nodes on the tree are Bayesian posterior probabilities (BP) (right) and ML bootstrap supports (BS) (left), respectively. (C) Morphological characters of *Uncaria*. The fruit pedicel (a), interfloral bracteole (b), and corolla tube length (c) of *Uncaria* species are mapped onto the phylogenetic tree. (D) Comparative genomic features, including genome size, LSC size and total SSR counts.

Complete circular plastomes of *Uncaria* have a typical quadripartite structure (Fig. 2A). Alignment of 73 shared plastid CDS yielded a phylogenetic matrix of 66,329 bp. Phylogenetic reconstruction indicated that *Uncaria* is a well-supported monophyletic group (BP = 1, BS = 99, Fig. 2B) with four major clades, while *Neolamarckia* and *Nauclea* formed a clade (BP = 1, BS = 98) being sister to *Uncaria* (BP = 1, BS = 100). Clade I consists of *Uncaria* species distributed in East Asia. Clade II forms a well-supported sister clade to Clade I (BP = 1, BS = 98) and consists of species distributed between tropical Asia and Oceania. These two clades are sister to Clade III, which consists of two tropical Asian species, *U. sessilifructus* Roxb. and *U. laevigata* Wal. ex G. Don (BP = 1, BS = 100). Clade IV, the most basal clade, consists of five species: *U. africana* G. Don, *U. angolensis* (Havil.) Welw. ex Hutch. & Dalziel, *U. donisii* E.M.A. Petit, *U. guianensis*, and *U. tomentosa*. Of these, the first three tropical African species formed a subclade and were sisters to the other two tropical American endemics.

Comparative analysis of plastomes revealed that the overall genome and LSC region sizes were larger in species from Clade IV than in species from other *Uncaria* clades (Fig. 2D). Interestingly, there was less SSR polymorphism in species from Clade IV; in addition, the majority of species in Clade IV and Clade I lacked pentanucleotide SSR (Table S9). Notably, specific SSR motifs were identified in some species, e.g., *U. callophylla* Blume ex Korth. had unique TTAC, TTTCT, and AAATAG motifs, and *U. tomentosa* held exclusive AAG and AAT motifs. The pentanucleotide AGAAT motif was detected in *Neolamarckia* and *Nauclea*, but absent in all examined *Uncaria* taxa (Table S9).

### 3.2 Divergence time estimation and diversification

**Fig. 3:**
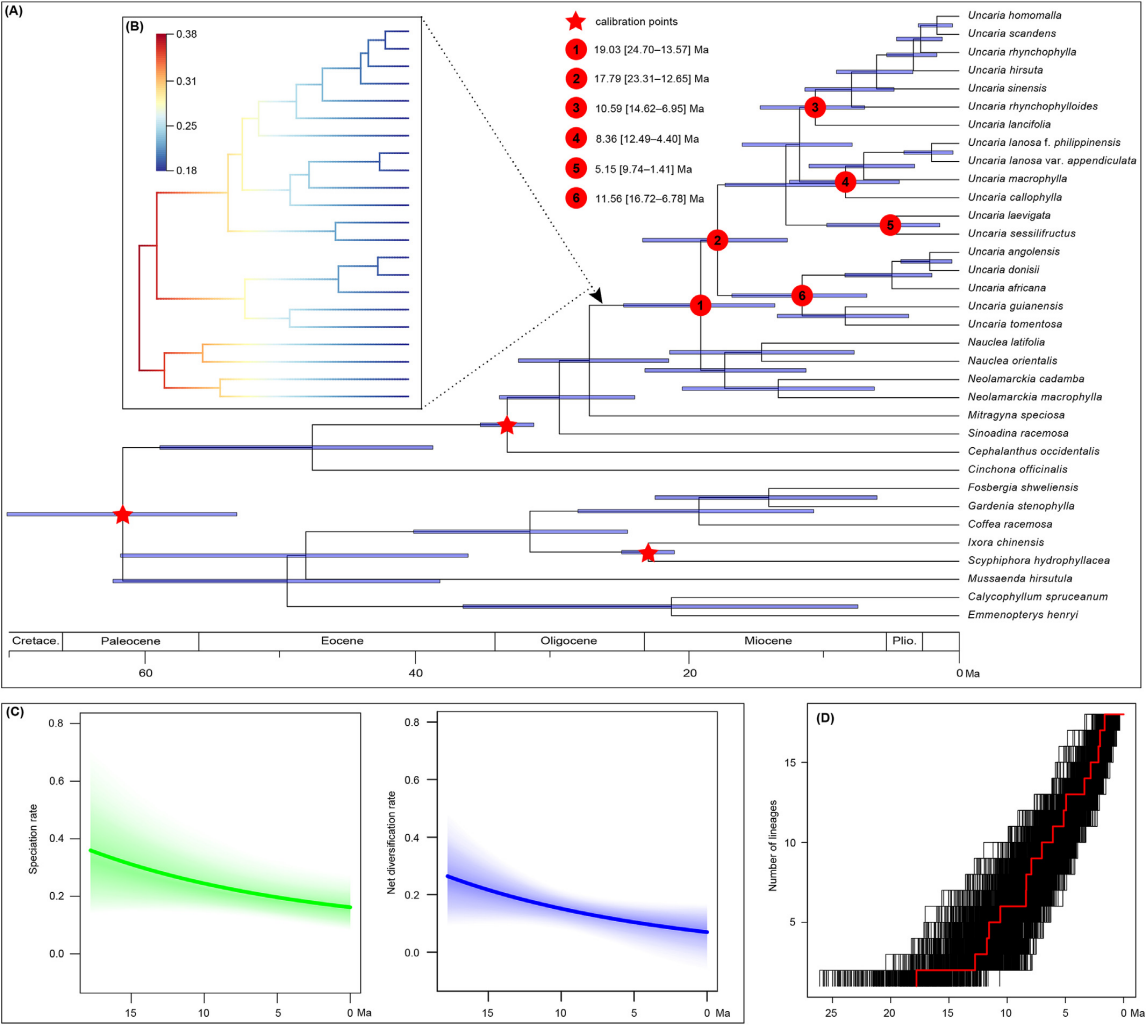
Diversification dynamics of *Uncaria*. (A) Chronogram of *Uncaria* derived from the MCC tree based on BEAST analyses with normal constraints. The blue bars indicate 95% HPD intervals of the age estimates. The red star represents fossil calibration nodes. Circles with numbers indicate significant nodes (nodes 1–6), with the estimated ages showing on the left. (B) Phylorate plot of *Uncaria* and its sister taxa inferred by BAMM analysis. (C) Temporal variation in speciation (green) and net diversification (blue) rates of *Uncaria*. Coloured polygon denotes a 95% confidence interval of rates. (D) Lineage-through-time plots showing the cumulative number of lineages over time for *Uncaria*. Red denotes the maximum clade credibility tree.

BEAST analyses employing different calibration protocols (i.e., normal, exponential, or uniform priors) yielded consistent estimates (Table S10). BEAST chronograms based on normal constraints indicated that *Uncaria* originated around 19.03 Ma in the early Miocene (95% higher posterior density [HPD] interval: 24.70–13.57 Ma, node 1 in Fig. 3A). The crown age of Clades I, II, and IV were estimated to be 10.59, 8.36, and 11.56 Ma, respectively (95% interval: 14.62–6.95, 12.49–4.40, 16.72–6.78 Ma; nodes 3, 4, and 6 in Fig. 3A). The crown age of the Clade III was dated to 5.15 Ma (95% HPD: 9.74–1.41 Ma, node 5 in Fig. 3A).

Bayesian analysis of macroevolutionary mixtures indicated that most *Uncaria* and their close relatives underwent early diversification but slowed towards the present, with no significant shift in diversification rate detected (Fig. 3B). The rate-through-time plots also showed a gradual decline in the global speciation and net diversification rates of *Uncaria* (Fig. 3C). LTT plots also showed similar results to Bayesian analysis of macroevolutionary mixtures for *Uncaria*, suggesting a faster lineage accumulation in the early stage but subsequently slowed down (Fig. 3D).

### 3.3 Ancestral range reconstruction

**Fig. 4:**
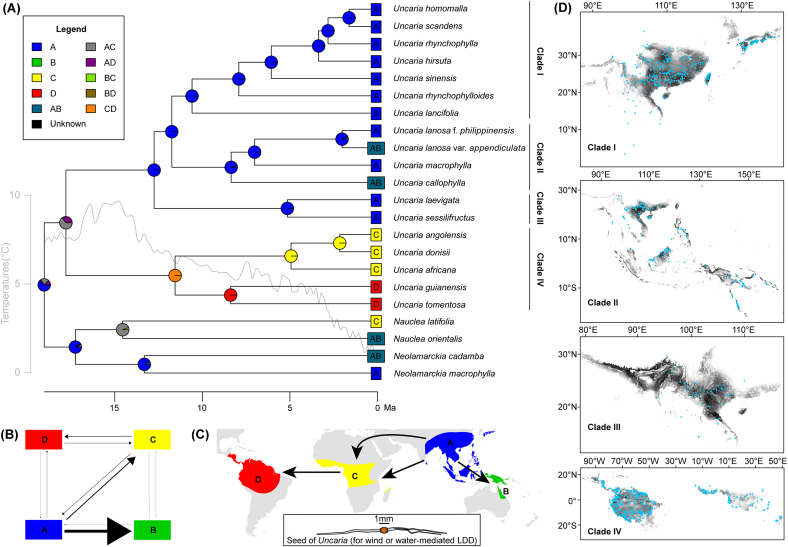
Biogeographical reconstruction of *Uncaria*. (A) Ancestral range reconstruction of *Uncaria* based on four regions, obtained using the DIVALIKE and constraint M1 models in BioGeoBEARS. Four regions were defined as follows: A represents Asia, B represents tropical Oceania, C represents tropical Africa, and D represents tropical America. A trend line of paleotemperatures (Zachos et al., 2008) was drawn using the smooth.spline function in R. (B) Historic exchanges among four defined regions for *Uncaria* and the closest sister taxa based on the biogeographical reconstruction. Arrow line thickness corresponds to the mean event counts based on the results of 100 BSMs. (C) Most likely dispersal routes of *Uncaria*. Seed belonged to the *Uncaria angolensis* and was drawn according to a seed from the voucher specimen of Deng2650. (D) Geographical distributions of four clades of *Uncaria*. Blue points represent the occurrences of all species within the clades, and black shades correspond to occurrence possibility as predicted by MaxEnt.

Both biogeographical DIVALIKE models, with constraints (M0 and M1), yielded similar results (Fig. 4 and Fig. S2). Here, we primarily refer to the results from the M1 model, as it better fits the data than does the unconstrained M0 model (log-likelihood, namely LNL: −24.88 versus −27.19, respectively). The M1 model had an extinction rate (e) of 1.0e^−12^ events/Ma and an anagenetic dispersal rate (d) of 0.021 events/Ma for *Uncaria* and its close allies. After 100 BSM runs, our model predicted 7.18 ± 0.46 anagenetic dispersal events, with the most frequent route from Asia to tropical Oceania (54.87 % of all dispersal events; Fig. 4B, Tables S11 and S12). The model also indicated that the major lineage source of tropical Oceania and Africa was Asia, while tropical America was a notable recipient of lineages from tropical Africa. The model also indicated that vicariance events were less frequent (3.63 ± 0.68; Fig. 4B, Table S11). The stem group of *Uncaria* was inferred to originate in Asia (Fig. 4A and C). Furthermore, the ancestral region for *Uncaria*’s crown was inferred to be Asia and tropical Africa (AC, Fig. 4A). The ancestral ranges of crown Clades I, II and III were also predicted to be Asia, whereas the ancestral range of crown Clade IV was predicted to have occurred in tropical Africa and America.

### 3.4 Niche modelling and ecological niche evolution of *Uncaria*

**Fig. 5:**
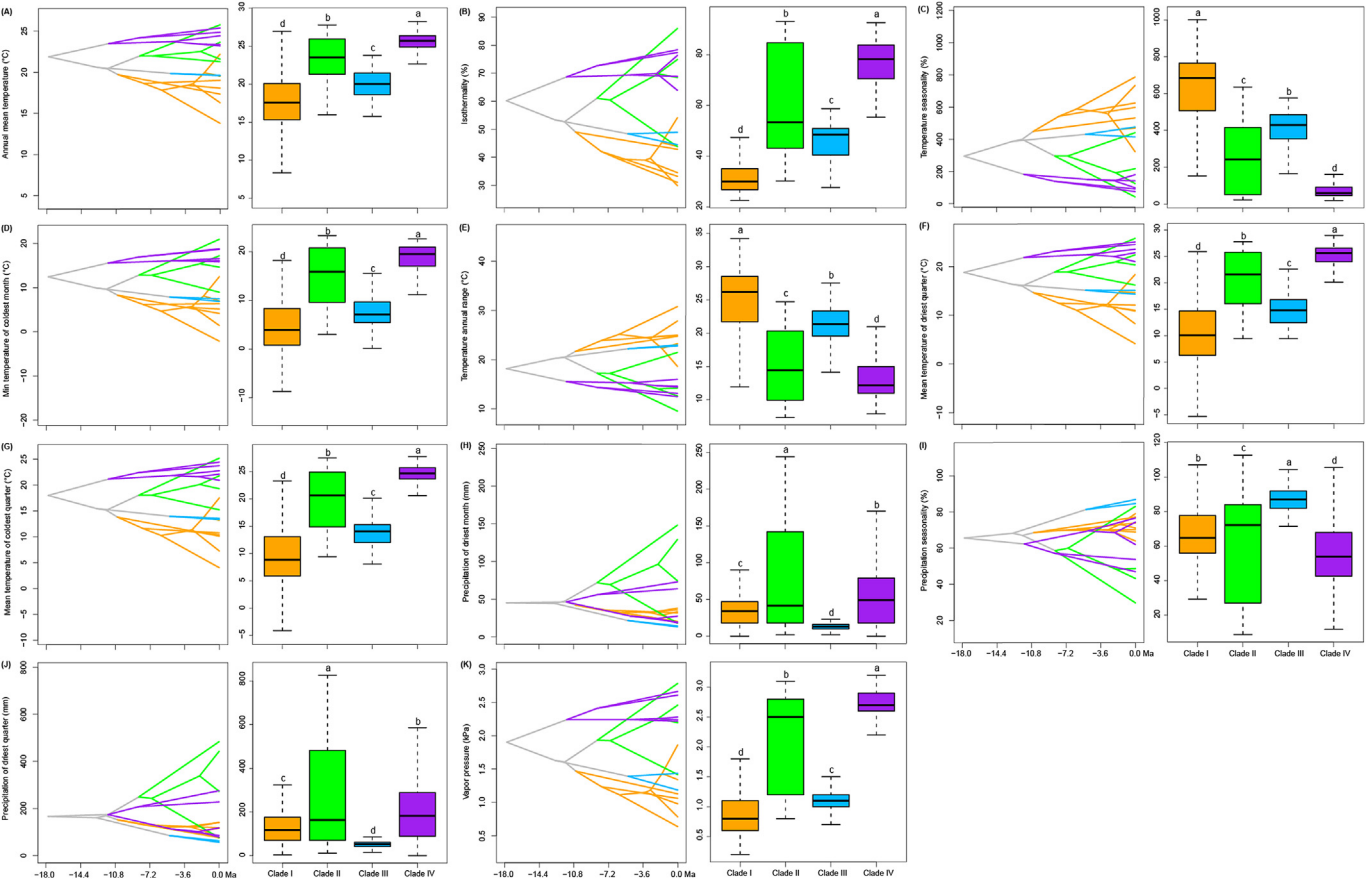
Ecological niche evolution in *Uncaria*. Ancestral niche breadth reconstruction and niche breadth comparison (A–K) of 11 factors for *Uncaria*. These factors were selected from 27 factors by comparing their niche breadths among four clades using Turkey HSD, with significant differences at the *P* < 0.05 level. Colours represent the four clades of this genus.

Occurrences and niche modelling in *Uncaria* are mainly pantropical (Fig. 4D). Clades I, II, and III occur mainly in East Asia, tropical Asia to Oceania, and tropical Asia, respectively, whereas Clade IV occurs only in tropical Africa and America. Environmental factors differed significantly between the four *Uncaria* clades, including seven temperature factors (annual mean temperature, bio1; isothermality, bio3; temperature seasonality bio4; min temperature of coldest month, bio6; temperature annual range, bio7; mean temperature of driest quarter, bio9; and mean temperature of coldest quarter, bio11), three precipitation factors (precipitation of driest month, bio14; precipitation seasonality, bio15; and precipitation of driest quarter, bio17), and vapor pressure (*P* < 0.05, Fig. 5A–K). Most temperature-related indices and vapor pressure were highest in Clade IV and lowest in Clade I, except for temperature seasonality and annual temperature range (*P* < 0.05, Fig. 5A–G). The historical reconstruction of ecological niches in *Uncaria* indicates that the temperature niche breadth began to gradually diverge in the early Miocene, while both temperature and precipitation niche breadths exhibited significant differentiation starting from the late Miocene (Fig. 5A–K).

## 4 Discussion

### 4.1 Two lineages and four clades of *Uncaria*

With samples from tropical Africa and tropical Asia to Oceania, we reconstructed the most comprehensive and robust phylogeny of *Uncaria* to date. Compared to previous studies (Zhang et al., 2015; Zhu et al., 2018; Liu et al., 2023), our phylogenetic results, which are based on 73 plastid protein-coding sequences from 29 accessions of *Uncaria* (16 accessions of this genus have been newly sequenced), obtained higher resolution and revealed four well-defined clades within *Uncaria* (Fig. 2B). Clades I–III and Clade IV, which we define as the Asia–Oceania and Afro–Neotropical lineages, are distinguished by their distribution, plastome size, morphology, and habitat (Fig. 4D). The Afro–Neotropical lineage has larger genome and LSC region size, associated with the extension of the *petN-psbM* and *rps16-trnQ* (UUG) intergenic regions (Fig. 2D). Only two species within the Afro–Neotropical lineage possess a corolla tube shorter than 5 mm. Furthermore, the Afro–Neotropical lineage tends to occur in warmer habitats (Fig. 2, Fig. 4). Our designation of an Afro–Neotropical lineage is consistent with the Afro–Neotropical clade of *Uncaria* proposed by Löfstrand et al. (2014), which was based on four plastid markers (*ndhF*, *rbcL*, *rps16*, and *trnT-F*) and two nuclear markers (nrETS and nrITS). Notably, the biogeographic pattern of these two lineages aligns with the two major tropical floristic regions of the world—American–African and Indo–Pacific—as defined in the phylogenetic classification by Slik et al. (2018).

Our phylogenetic topology also showed high congruence regarding the grouping of specific species in Clade I (*Uncaria* *lancifolia* Hutch. to *U. homomalla* Miq., Fig. 2B) in East Asia, which aligns with the findings of a previous plastid phylogenetic framework of *Uncaria* (Dai et al., 2023). Clade I tends to exhibit a stronger preference for colder environments compared to the other three clades (Fig. 5). We also found Clade II consists of *U. callophylla*, *U. macrophylla* Wall., and two infraspecific taxa of *U. lanosa*. Species in Clade II have fruit pedicels and lack interfloral bracteoles, which are distributed between tropical Asia and Oceania. Clade III consists of *U. laevigata* and *U. sessilifructus*, which do not have fruit pedicels and interfloral bracteoles present in species from tropical Asia (Ridsdale, 1978; Chen and Taylor, 2011) (Fig. 2, Fig. 4D). The sampling size and plastome data used in this study have some limitations, e.g., plastome data may be skewed by maternal inheritance. Given the increasing availability of molecular data, future studies could integrate a broader set of nuclear genes, such as deeply sequenced Angiosperms353 single-copy nuclear genes (Zhang et al., 2022; Chen et al., 2023; Kipkoech et al., 2025), to further consolidate intrageneric relationships.

### 4.2 A case for a young origin of liana in pantropical forests

With two fossils and a secondary calibration, our analyses dated the origins of stem-lineage *Uncaria* at ca. 19.03 Ma (95% HPD: 24.70–13.57 Ma, node 1 in Fig. 3A), which was largely close to the age estimated by Nie et al. (2013). However, the stem age of this genus differed from the age in Yang et al. (2022) due to inconsistencies in the closest sister taxa of *Uncaria* based on several nuclear and plastid markers. In comparison to the origin time of other liana taxa with the PID pattern, such as *Cissus* (Vitaceae; Liu et al., 2013) and *Paederia* (Rubiaceae; Nie et al., 2013), the early Miocene origin of *Uncaria* is relatively young, providing an example of a recent origin in the pantropical realm. Our ancestral range reconstruction of *Uncaria* suggested the Asian origin for this genus (Fig. 4A and C), aligning with the high species diversity of *Uncaria* in this region. Similarly, other cases on liana taxa with PIDs have indicated that Asia may have served as a cradle for the lianas in pantropical forests, including Cucurbitaceae (Schaefer et al., 2009), Menispermaceae (Wang et al., 2012), and *Paederia* (Rubiaceae; Nie et al., 2013).

### 4.3 Diversification of *Uncaria* driven largely by climate

BAMM analysis revealed that the stem group of *Uncaria* had a diversification rate of 0.38 species per million years (species/Ma) at *ca*. 19.03 Ma (Fig. 3B). This diversification rate was higher than rates documented in Menispermaceae (0.05/0.03 species/Ma; Wang et al., 2012) and 17 angiosperm clades (ca. 0.12 species/Ma; Xing and Ree, 2017), but was lower than rates in many fast plant radiations including alpine bamboos of the Hengduan Mountains (0.75 species/Ma, Poaceae; Ye et al., 2019) and Andean bellflowers (1.83 species/Ma, Campanulaceae; Lagomarsino et al., 2016). The subsequent diversification of *Uncaria* (17.79 Ma, node2, Fig. 3A and B) remained rapid until the occurrence of the major clades during the late Miocene, which is also supported by the result of LTT (Fig. 3D). This rapid early diversification of *Uncaria* was likely associated with a warming phase peaking in the Middle Miocene Climatic Optimum (MMCO, *ca*. 17–15 Ma; Zachos et al., 2001).

Niche reconstruction indicates that *Uncaria* clades began to emerge progressively during the early Miocene (Fig. 5A–G). Our finding that temperature-related factors differed significantly between *Uncaria* clades indicates that the early rapid diversification of *Uncaria* may have been facilitated by these environmental factors (Fig. 3A, B and D). During global cooling of the late Miocene (Zachos et al., 2001), a pronounced divergence in temperature niche breadth occurred within the genus (Fig. 5A–G). This likely reflects an adaptation of certain lineages to cooler environments, while others remained restricted to warmer conditions. Ultimately, this divergence may have promoted the diversification of cold-tolerant lineages—particularly Clade I, which is distributed in relatively high latitudes—and heat-adapted lineages, especially Clade IV, found in relatively low latitudes (Fig. 4D). This diversification is further corroborated by the divergence in niche breadth associated with vapor pressure, a factor closely linked to temperature (Fig. 5K). Among the three precipitation-related variables, a marked divergence in niche breadth emerged in Clades II and IV of *Uncaria* since the late Miocene (Fig. 5H–J), which was likely influenced by local geological history. For example, the precipitation niche breadths of species in Clade II began to rapidly diverge from its crown group at ca. 8.36 Ma (Figs. 2A and 4H–J), coinciding with a major period of the uplift of the Qinghai-Tibet Plateau, around 8–7 Ma (Harrison et al., 1992; Guo et al., 2002; Spicer et al., 2003) and the onset of the Indian and East Asian monsoons, about 9–8 Ma (An et al., 2001). The divergences of these precipitation niche breadths likely promoted the subsequent diversification of this clade, especially for its tropical Asian taxa.

### 4.4 Role of the long-distance dispersal in the formation of PID pattern

Biogeographical stochastic mapping of *Uncaria*, *Nauclea* and *Neolamarckia* showed that the most frequent dispersal events of the PIDs were from Asia to tropical Oceania (54.87% of all dispersal events, Fig. 4B, Tables S11 and S12). These findings are consistent with a previous meta-analysis that found migration frequences from tropical Asia to Australasia were more than two times that of the reverse since the middle Eocene, except for terminal migrations (Zhang et al., 2023). In this study, *Uncaria* dispersal events were mostly found after the middle Miocene (Fig. 4A), supporting the hypothesis that the main migrations between Asia and tropical Oceania occurred after 15 Ma (Zhang et al., 2023; Chen et al., 2024), when Australasia had already collided with Southeast Asia (ca. 25 Ma) (Hall, 2002, 2009). Notably, the seeds of *Uncaria* are small and usually display a pair of long wings (Ridsdale, 1978; Chen and Taylor, 2011). This trait suggests a remarkable adaptation to wind or water dispersal and likely contributed to the stepping-stone dispersal events from Asia to tropical Oceania (Fig. 4A, B, and C). Studies have shown that 49% of immigration events from tropical Asia to Australasia after the middle Miocene consisted of taxa with abiotic dispersal associated with non-fleshy fruits, e.g., capsules with small seeds, capsules with winged seeds, samaras, follicles with winged seeds, and pods (Zhang et al., 2023). In contrast, the predominant mode of migration between tropical Asia and tropical Oceania has been shown to be biotic dispersal, contributing to up to 72% of migration events across 29 taxa (Zhang et al., 2023). Animals species that have been found to promote seed dispersal in *Nauclea* and *Neolamarckia* include fruit bats, *Pteropus giganteus*, *Cynopterus sphinx* (for *Neolamarckia*; Mahandran et al., 2021) and proboscis monkeys (for *Nauclea*; Thiry et al., 2019).

Ancestral range reconstruction indicated that the ancestors of *Uncaria* in tropical Africa originated in Asia during 19.03–17.79 Ma. The ancestors of tropical American taxa were inferred to come mainly from Asia and tropical Africa during 17.79–11.56 Ma (Fig. 3, Fig. 4A and C). These findings raise the question of how *Uncaria* were distributed in tropical Africa and America. One possible explanation for this distribution is vicariance via tectonic movement and boreotropical migration during the Paleocene-Eocene Thermal Maximum (Givnish and Renner, 2004; Wei et al., 2015; Viruel et al., 2016; Selvatti et al., 2022), however, *Uncaria* originated and diversified after these events. Instead, we speculate that this genus may have migrated from Asia to tropical Africa through land connections formed between Southwest Asia and tropical Africa in the early to mid-Miocene (Rögl, 1998; Popov et al., 2004; Yu et al., 2014) during the Middle Miocene Climate Optimum (*ca*. 17–15 Ma) (Zachos et al., 2001). The Bering Land Bridge, which was available to terrestrial biota from the early Paleocene to 7.4–4.8 Ma (Tiffney and Manchester, 2001), has often been considered a migration highway for temperate taxa (Wen et al., 2010; Huang et al., 2021). However, the presence of several thermophilic taxa, such as *Dioscorea* (Dioscoreaceae; Viruel et al., 2016), *Sageretia* (Rhamnaceae; Yang et al., 2019) and Urticeae (Urticaceae; Huang et al., 2019), which were also reported to have migrated via the Bering Land Bridge during this period, particularly in the Miocene, suggests that this route cannot be ruled out in the dispersal of *Uncaria*, despite the lack of direct fossil evidence.

One alternative explanation supported by the small-winged seeds of *Uncaria* species is that long-distance dispersal into tropical Africa and America was mediated by wind or water. If so, the route of dispersal was most likely stepwise, from Asia to tropical Africa, followed by a subsequent dispersal to tropical America. This explanation aligns with an increasing body of biogeographical studies indicating that such dispersal events were driven by ocean currents, notably the Indian Ocean currents for taxa such as *Cycas* (Cycadaceae; Liu et al., 2021), *Paederia* (Nie et al., 2013), and Urticeae (Huang et al., 2019), and the Atlantic currents for taxa such as *Barleria oenotheroides* (Acanthaceae; Martín-Bravo and Daniel, 2016), *Guibourtia* (Fabaceae; Tosso et al., 2018), and *Manilkara* (Sapotaceae; Armstrong et al., 2014). Studies have also shown that during the Miocene Africa was connected to the Neotropics, and East Africa to South India/Southeast Asia, by transoceanic rafting along the North and South Equatorial Counter Currents (Houle, 1998). Our finding that wind speed was a component of *Uncaria* ecological niches (Table S8) also implies that wind-mediated long-distance dispersal is at least partly responsible for the current distribution of the genus. This explanation would be consistent with studies that have shown numerous plant lineages with similar PID patterns, such as *Paederia* (Nie et al., 2013) and Urticeae (Wu et al., 2018; Huang et al., 2019), followed similar long-distance dispersal routes (Morley, 2003). Thus, together with other studies, our findings would highlight the importance of long-distance dispersal in shaping tropical biogeographical patterns.

## 5 Conclusions

In this study, we reconstructed a comprehensive phylogeny of *Uncaria*, inferred its spatiotemporal evolution, and tested hypotheses accounting for its PID pattern. *Uncaria* likely originated in Asia during the early Miocene before diverging into two lineages (and four clades) during its dispersal to other continents outside of Asia in the pantropics, mainly through long-distance dispersal. The early rapid diversification of *Uncaria* was associated with the Middle Miocene Climate Optimum, and its following diversification was linked to the divergences of ecological niches, especially climatic factors. Taken together, our results suggest long-distance dispersal and climate factors have jointly contributed to the formation of the PIDs of *Uncaria*. Further phylogenetic studies on *Uncaria* should integrate a more comprehensive set of nuclear genes, such as AGS353 single-copy nuclear genes, along with denser species sampling—particularly of Oceania-endemic species—to further uncover the evolution of this genus.

## CRediT authorship contribution statement

**Xian-Han Huang:** Writing – original draft, Project administration, Funding acquisition, Investigation, Visualization, Methodology, Formal analysis, Conceptualization. **Jing-Yi Peng:** Writing – original draft, Visualization, Methodology, Formal analysis, Data curation. **Nan Lin:** Writing – original draft, Funding acquisition, Methodology, Formal analysis, Software. **Jian Liu:** Writing – review & editing, Methodology. **Jun-Tong Chen:** Investigation, Resources, Software. **Qun Liu:** Investigation, Resources, Software. **Xin-Jian Zhang:** Investigation, Resources, Software. **Quan-Sheng Fu:** Investigation, Software. **Peng-Rui Luo:** Investigation, Resources. **Zhi-Yu Wang:** Investigation, Resources. **Shiou Yih Lee:** Writing – review & editing. **Qiang Zhou:** Writing – review & editing. **Hang Sun:** Writing – review & editing, Project administration, Funding acquisition, Resources, Supervision, Methodology, Investigation, Conceptualization. **Tao Deng:** Writing – review & editing, Project administration, Funding acquisition, Resources, Supervision, Methodology, Investigation, Conceptualization.

## Data availability

The sequences used in this study are available at NCBI database and the accession numbers are presented in Table S1 and Table S2.

## Declaration of competing interest

The authors declare that they have no known competing financial interests or personal relationships that could have appeared to influence the work reported in this paper.
