# Supplementary material for: Climatic niche divergence and long-distance dispersal contributed to the pantropical intercontinental disjunctions of a liana lineage (Uncaria, Rubiaceae)
Source: Plant Divers. 2025 May 9;47(5):772–83. doi: 10.1016/j.pld.2025.05.001 (PMC12496531; doi:10.1016/j.pld.2025.05.001)
Supplement: Multimedia component 1 [file mmc1.docx]

Climatic niche divergence and long-distance dispersal contributed to the pantropical intercontinental disjunctions of a liana lineage (*Uncaria*, Rubiaceae)

**This** **Word file includes:**

Figs. S1 to S3,

Tables S1 to S12.


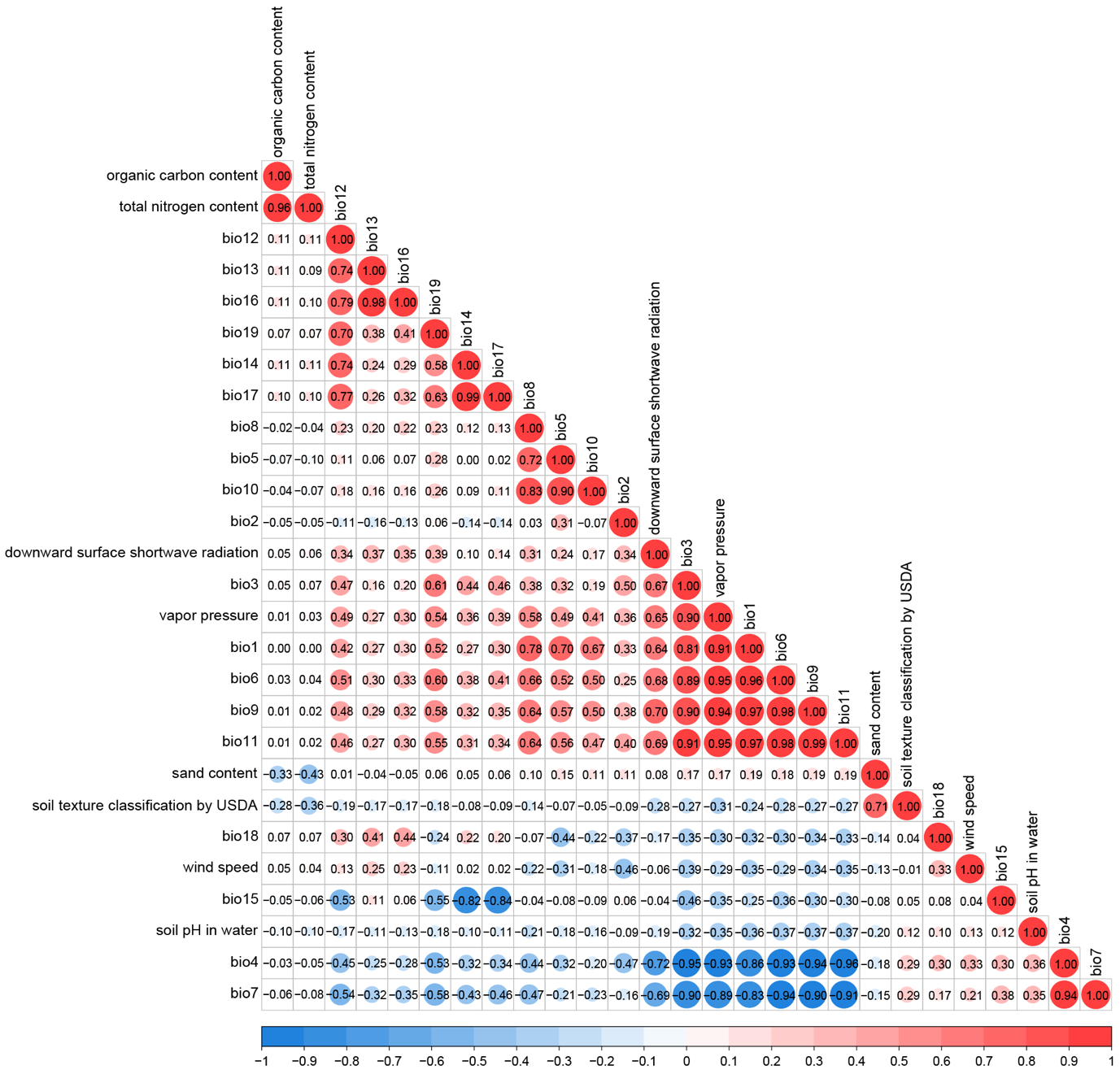


**Fig. S1.** Correlation heat maps of 27 climatic factors and their contribution to the first PCA in *Uncaria*. Numbers within the circle represent the correlation coefficient. Blue circles represent negative correlation, and red circles represent positive correlation. Size of the circle is directly proportional to its correlation.

Abbreviations: bio1, annual mean temperature; bio2, mean diurnal range (mean of monthly (max temperature–min temperature)); bio3, isothermality (bio2/bio7×100); bio4, temperature seasonality (standard deviation×100); bio5, maximum temperature of the warmest month; bio6, minimum temperature of the coldest month; bio7, temperature annual range (bio5–bio6); bio8, mean temperature of the wettest quarter; bio9, mean temperature of the driest quarter; bio10, mean temperature of the warmest quarter; bio11, mean temperature of the coldest quarter; bio12, annual precipitation; bio13, precipitation of the wettest month; bio14, precipitation of the driest month; bio15, precipitation seasonality (coefficient of variation); bio16, precipitation of the wettest quarter; bio17, precipitation of the driest quarter; bio18, precipitation of the warmest quarter; bio19, precipitation of the coldest quarter.

**
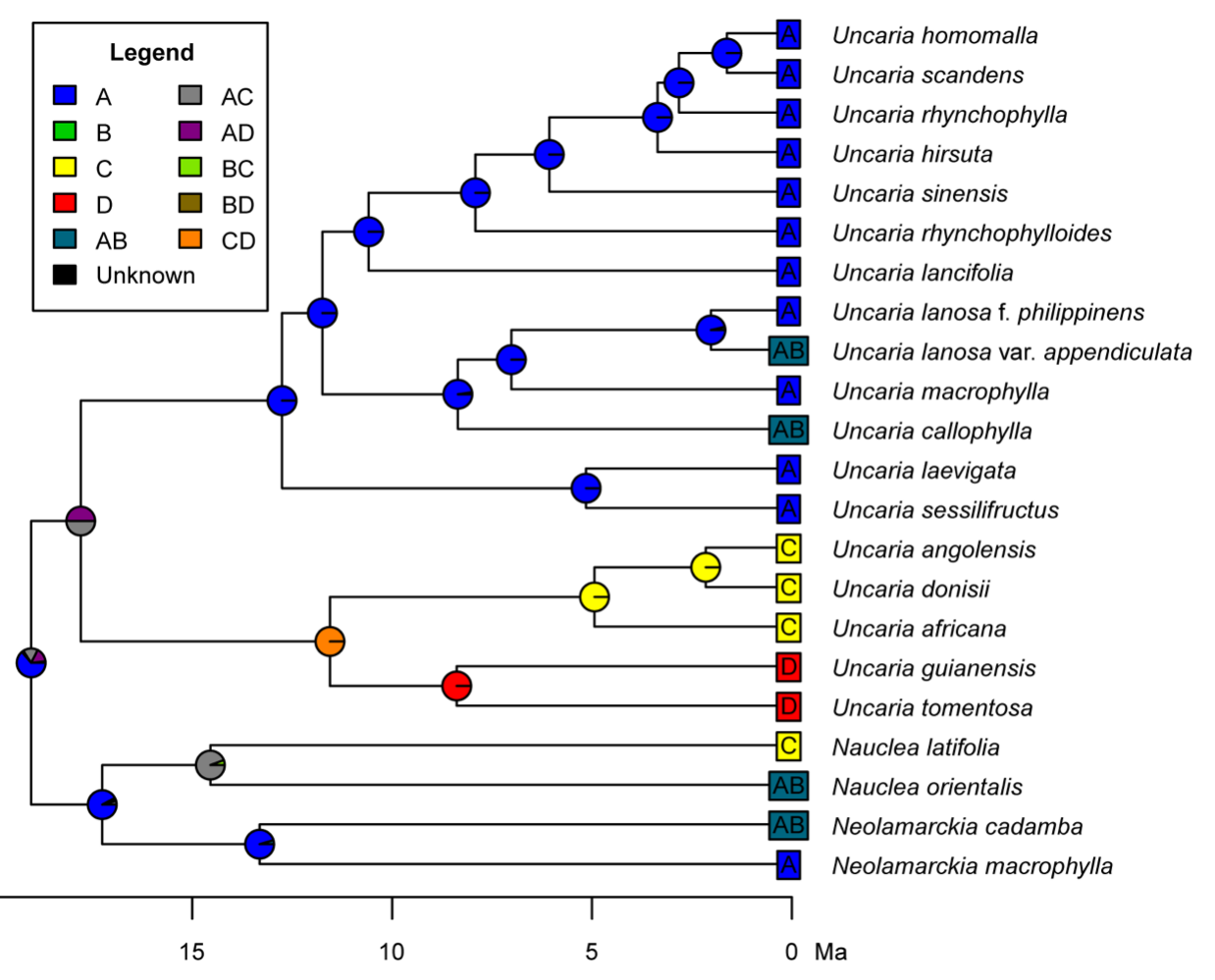
**

**Fig. S2.** Ancestral area reconstruction of *Uncaria* using the DIVALIKE and unconstrained M0 models in BioGeoBEARS.


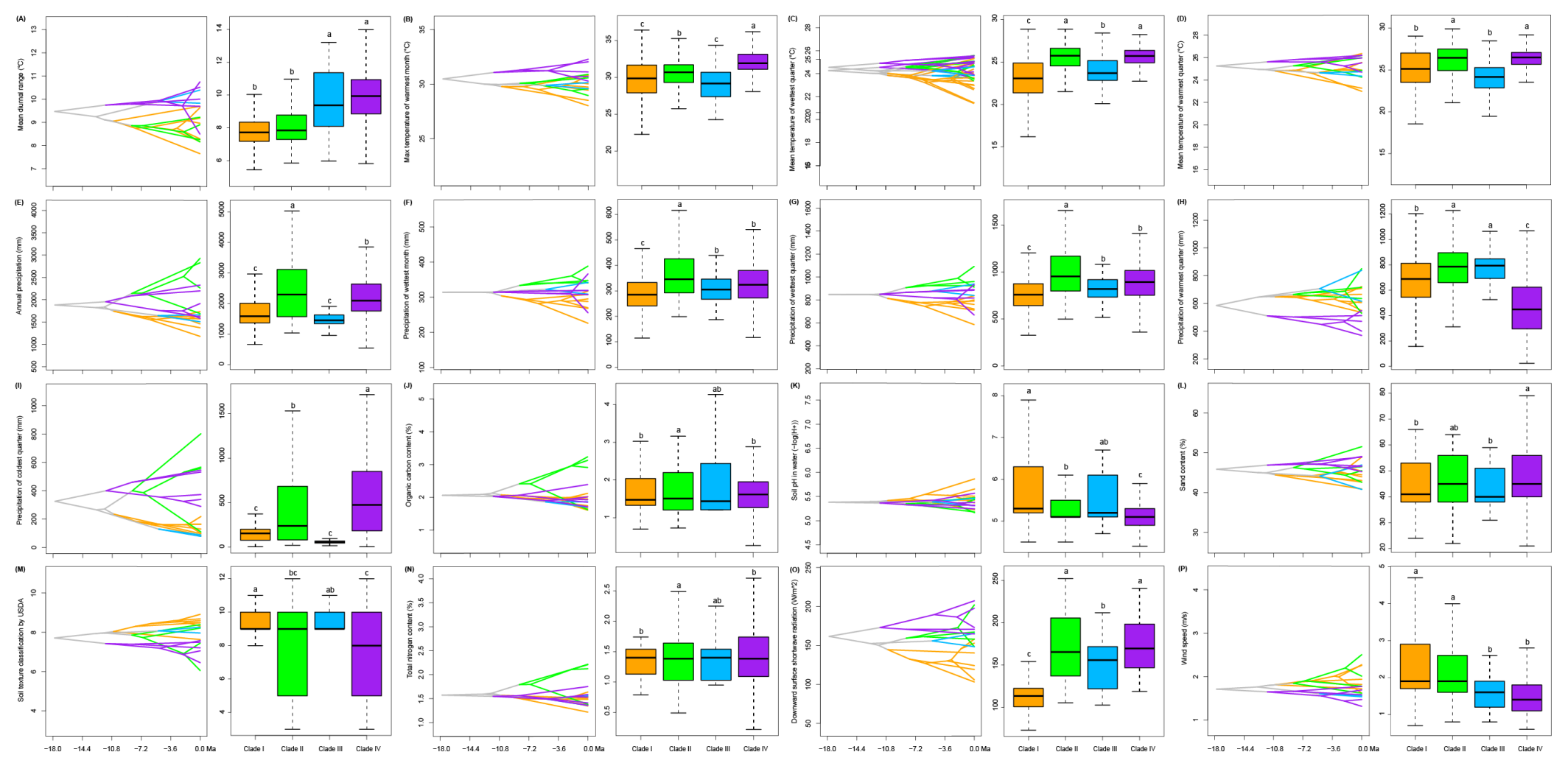


**Fig. S3.** Climatic niche evolution of *Uncaria* in addition to Fig. 5.

**Table S1.** Sample information, GenBank accessions, and characteristics of newly assembled plastid genomes for *Uncaria* and its sister taxa.

| **Number** | **Taxon name** | **Source** | **Collection locality** | **Collection or voucher number** | **Sequencing code** | **Size of Total / LSC / SSC / IR (bp)** | **GC content of Total / LSC / SSC / IR (%)** | **Number of genes (CDS / tRNA / rRNA)** | **GenBank accession** |
| --- | --- | --- | --- | --- | --- | --- | --- | --- | --- |
| 1 | *Uncaria rhynchophylla* | Herbarium of Kunming Institute of Botany, China | Kunming, Yunnan, China | Huangxh001 | PJY12 | 154,869 / 85,482 / 18,045 / 25,671 | 37.6 / 35.6 / 31.8 / 43.2 | 131 (86 / 37 / 8) | PP093062 |
| 2 | *Uncaria homomalla* | Herbarium of Kunming Institute of Botany, China | Lvchun, Yunnan, China | DengHLS168 | PJY26 | 154,829 / 85,446 / 18,055 / 25,664 | 37.6 / 35.5 / 31.7 / 43.2 | 131 (86 / 37 / 8) | PP350072 |
| 3 | *Uncaria hirsuta* | Herbarium of Kunming Institute of Botany, China | Malipo, Yunnan, China | Deng10673 | HXH74 | 154,860 / 85,469 / 18,063 / 25,664 | 37.6 / 35.5 / 31.7 / 43.2 | 131 (86 / 37 / 8) | PP093052 |
| 4 | *Uncaria hirsuta* | Herbarium of Kunming Institute of Botany, China | Xishuangbanna, Yunnan, China | Deng14220 | HXH79 | 154,776 / 85,441 / 18,005 / 25,665 | 37.6 / 35.6 / 31.7 / 43.2 | 131 (86 / 37 / 8) | PP093054 |
| 5 | *Uncaria sinensis* | Herbarium of Kunming Institute of Botany, China | Jiujiang, Jiangxi, China | TanCM1637 | PJY18 | 154,881 / 85,489 / 18,088 / 25,652 | 37.6 / 35.6 / 31.6 / 43.2 | 131 (86 / 37 / 8) | PP093067 |
| 6 | *Uncaria rhynchophylloides* | Herbarium of Kunming Institute of Botany, China | Fangchenggang, Guangxi, China | GX495 | PJY17 | 155,002 / 85,608 / 18,120 / 25,637 | 37.6 / 35.5 / 31.6 / 43.2 | 131 (86 / 37 / 8) | PP093066 |
| 7 | *Uncaria lancifolia* | Herbarium of Kunming Institute of Botany, China | Gongshan, Yunnan, China | Sunjun001 | PJY05 | 154,792 / 85,288 / 18,200 / 25,652 | 37.6 / 35.6 / 31.5 / 43.2 | 131 (86 / 37 / 8) | PP093060 |
| 8 | *Uncaria lancifolia* | Herbarium of Kunming Institute of Botany, China | Cangyuan, Yunnan, China | No. 5309270854 | PJY15 | 154,795 / 85,308 / 18,169 / 25,659 | 37.6 / 35.5 / 31.5 / 43.2 | 131 (86 / 37 / 8) | PP093068 |
| 9 | *Uncaria macrophylla* | Herbarium of Kunming Institute of Botany, China | Simao, Yunnan, China | Liu Ende et al. 4056 | PJY13 | 155,146 / 85,757 / 18,059 / 25,665 | 37.5 / 35.4 / 31.5 / 43.2 | 131 (86 / 37 / 8) | PP093063 |
| 10 | Uncaria lanosa f. philippinensis | Herbarium of Kunming Institute of Botany, China | Manzhoulide, Taiwan, China | TW-2012244 | PJY04 | 154,955 / 85,576 / 18,067 / 25,656 | 37.5 / 35.4 / 31.5 / 43.2 | 131 (86 / 37 / 8) | PP093059 |
| 11 | Uncaria lanosa var. appendiculata | Queensland Herbarium, Australia | Cook, Queensland, Australia | AQ774615 | HXH115 | 154,998 / 85,598 / 18,066 / 25,667 | 37.5 / 35.4 / 31.5 / 43.2 | 131 (86 / 37 / 8) | PP093056 |
| 12 | *Uncaria laevigata* | Herbarium of Kunming Institute of Botany, China | Jiangcheng, Yunnan, China | YiMuMK-159 | PJY14 | 155,233 / 85,770 / 18,139 / 25,662 | 37.6 / 35.5 / 31.5 / 43.2 | 131 (86 / 37 / 8) | PP093064 |
| 13 | *Uncaria angolensis* | Herbarium of Kunming Institute of Botany, China | Kenya, Africa | Deng2650 | PJY10 | 155,590 / 86,089 / 18,113 / 25,694 | 37.5 / 35.4 / 31.5 / 43.2 | 131 (86 / 37 / 8) | PP093061 |
| 14 | *Uncaria callophylla* | Queensland Herbarium, Australia | Cook, Queensland, Australia | AQ1009514 | HXH118 | 154,850 / 85,438 / 18,096 / 25,658 | 37.5 / 35.4 / 31.5 / 43.2 | 131 (86 / 37 / 8) | PP093057 |
| 15 | *Uncaria africana* | Missouri Botanical Garden, USA | Guinea, Africa | NO. 6177097 | PJY19 | 155,666 / 86,115 / 18,187 / 25,682 | 37.5 / 35.4 / 31.5 / 43.2 | 131 (86 / 37 / 8) | PP350069 |
| 16 | *Uncaria donisii* | Missouri Botanical Garden, USA | Gabon, Africa | NO. 6682771 | PJY20 | 155,671 / 86,133 / 18,148 / 25,695 | 37.5 / 35.4 / 31.6 / 43.2 | 131 (86 / 37 / 8) | PP350070 |
| 17 | *Neolamarckia cadamba* | Herbarium of Kunming Institute of Botany, China | Guangzhou, Guangdong, China | Huangxh002-1 | HXH82 | 155,000 / 85,881 / 17,851 / 25,634 | 37.6 / 35.4 / 31.6 / 43.2 | 131 (86 / 37 / 8) | PP093055 |
| 18 | *Nauclea latifolia* | Missouri Botanical Garden, USA | Gabon, Africa | NO. 6742831 | PJY23 | 155,335 / 85,948 / 18,091 / 25,648 | 37.6 / 35.4 / 31.6 / 43.2 | 131 (86 / 37 / 8) | PP350071 |
| 19 | *Nauclea orientalis* | Queensland Herbarium, Australia | Cook, Queensland, Australia | AQ906843 | HXH122 | 155,366 / 85,952 / 18,102 / 25,656 | 37.6 / 35.5 / 31.7 / 43.2 | 131 (86 / 37 / 8) | PP093058 |
| 20 | *Cephalanthus occidentalis* | The Harvard University Herbaria, USA | Massachusetts, America | RIB No. 2539 | HXH78 | 155,213 / 85,785/ 18,106 / 25,661 | 37.6 / 35.5 / 31.5 / 43.2 | 131 (86 / 37 / 8) | PQ557237 |

**Table S2.** GenBank accessions of the downloaded plastid genomes for *Uncaria* and its sister taxa.

| **Number** | **Taxon name** | **Genbank accession** |
| --- | --- | --- |
| 1 | *Uncaria homomalla* | OQ679828 |
| 2 | *Uncaria scandens* | ON243637 |
| 3 | *Uncaria rhynchophylla* 1 | MT991006 |
| 4 | *Uncaria rhynchophylla* 2 | OQ679830 |
| 5 | *Uncaria hirsuta* | OQ679827 |
| 6 | *Uncaria sinensis* | OQ679832 |
| 7 | *Uncaria rhynchophylloides* | OQ679831 |
| 8 | *Uncaria lancifolia* | OQ679829 |
| 9 | *Uncaria macrophylla* 1 | ON243636 |
| 10 | *Uncaria macrophylla* 2 | MZ869757 |
| 11 | *Uncaria sessilifructus* | ON243635 |
| 12 | *Uncaria guianensis* | OP794339 |
| 13 | *Uncaria tomentosa* | OP794340 |
| 14 | *Neolamarckia cadamba* | MG572117 |
| 15 | *Neolamarckia macrophylla* | MN877388 |
| 16 | *Sinoadina racemosa* | MW801112 |
| 17 | *Mitragyna speciosa* | KY085908 |
| 18 | *Cinchona officinalis* | MZ151891 |
| 19 | *Fosberaia shweliensis* (outgroup) | MT480075 |
| 20 | *Gardenia stenophylla* (outgroup) | OL517769 |
| 21 | *Coffea racemosa* (outgroup) | MW970412 |
| 22 | *lxora chinensis* (outgroup) | MZ221832 |
| 23 | *Scyphiphora hydrophyllacea* (outgroup) | MN390972 |
| 24 | *Mussaenda hirsutula* (outgroup) | MK203878 |
| 25 | *Emmenopterys henryi* (outgroup) | KY273445 |
| 26 | *Calycophyllum spruceanum* (outgroup) | OK326865 |

**Table 3.** Seven key characters of *Uncaria*. For fruit pedicel, interfloral bracteole, leaves hairs, corolla tube hairs and corolla lobe hairs, “Y” represents “present”, “N” represents “absent”, and “?” represents “unknown”. For corolla tube length, “Y” represents “>5mm”, “N” represents “<5mm”, and “?” represents “unknown”. The range of data measurements and corresponding voucher specimens or references are provided in parentheses. For stipule shape, “Y” represents “entire”, “N” represents “bifid”, and “?” represents “unknown”.

| **Number** | **Taxon** | **Sampling situation** | **fruit pedicel** | **interfloral bracteole** | **corolla tube length** | **leaves hairs** | **stipule shape** | **corolla tube hairs** | **corolla lobe hirs** |
| --- | --- | --- | --- | --- | --- | --- | --- | --- | --- |
| 1 | *U. hirsuta* | Sampled | N | Y | Y, (7–10.5mm, Flora of China) | Y | N | Y | Y |
| 2 | *U. homomalla* | Sampled | N | Y | Y, (5–8.5mm, Flora of China) | Y | N | Y | Y |
| 3 | *U. lancifolia* | Sampled | N | Y | Y, (9–12mm, Flora of China) | N | N | N | N |
| 4 | *U. rhynchophylla* | Sampled | N | Y | Y, (6–8mm, Ridsdale 1978) | N | N | N | N |
| 5 | *U. rhynchophylloides* | Sampled | N | Y | Y, (ca. 12 mm, Flora of China) | N | N | N | N |
| 6 | *U. scandens* | Sampled | N | Y | Y, (8–10mm, Flora of China) | Y | N | Y | Y |
| 7 | *U. sinensis* | Sampled | N | Y | Y, (7–8mm, Flora of China) | N | Y | N | Y |
| 8 | *U. callophylla* | Sampled | Y | N | Y, (8–10mm, Herbier Museum Paris: P03918019) | N | Y | Y | Y |
| 9 | *U. lanosa* | Sampled | Y | N | Y, (ca. 12mm, Flora of China) | Y | N | N | N |
| 10 | *U. macrophylla* | Sampled | Y | N | Y, (9–10mm, Flora of China) | Y | N | Y | Y |
| 11 | *U. laevigata* | Sampled | N | Y | Y, (7–10mm, Flora of China) | N | N | N | N |
| 12 | *U. sessilifructus* | Sampled | N | Y | Y, (6–10mm, Flora of China) | N | N | N | Y |
| 13 | *U. africana* | Sampled | Y | N | Y, (7–9.5mm, Herb. Horti Bot. Nat. Belg.: BR885904) | N | N | Y | Y |
| 14 | *U. angolensis* | Sampled | Y | N | Y, (8–9mm, The Natural History Museum London Department of Botany: BM000902815) | N | N | Y | Y |
| 15 | *U. donisii* | Sampled | Y | N | Y, (8–10mm, Herb. Horti Bot. Nat. Belg.: BR885864) | Y | N | Y | Y |
| 16 | *U. guianensis* | Sampled | Y | Y | N, (2.5–3.5mm, Ridsdale 1978) | N | Y | N | Y |
| 17 | *U. tomentosa* | Sampled | Y | Y | N, (3.5–5mm, Ridsdale 1978) | Y | Y | Y | Y |
| 18 | *U. acida* | Not sampled | Y | N | Y, (6–8mm, Royal Botanic Gardens Kew: K000729955) | ？ | N | Y | Y |
| 19 | *U. attenuata* | Not sampled | Y | N | Y, (8–9.5mm, Royal Botanic Gardens Kew: K000729999) | Y | Y | Y | Y |
| 20 | *U. barbata* | Not sampled | Y | N | Y, (8.5–10mm, Herb. Horti Bot. Nat. Belg.: BR532746) | Y | Y | Y | Y |
| 21 | *U. bernaysii* | Not sampled | Y | N | ？ | ？ | N | Y | Y |
| 22 | *U. borneensis* | Not sampled | Y | N | ？ | Y | ? | Y | Y |
| 23 | *U. canescens* | Not sampled | Y | N | Y, (7–9mm, Royal Botanic Gardens Kew: K000760046) | ？ | N | Y | Y |
| 24 | *U. cordata* | Not sampled | Y | N | Y, (10–13mm, Royal Botanic Gardens Kew: K001129423) | Y | N | Y | Y |
| 25 | *U. elliptica* | Not sampled | Y | N | Y, (9.5–12mm, Royal Botanic Gardens Kew: K001123046) | Y | Y | Y | Y |
| 26 | *U. gambir* | Not sampled | Y | N | Y, (10–12mm, Royal Botanic Gardens Kew: K001123036) | ？ | Y | Y | Y |
| 27 | *U. kunstleri* | Not sampled | Y | N | Y, (5.5–7mm, Singapore Botanic Gardens: SING0053110) | ？ | N | Y | Y |
| 28 | *U. longiflora* | Not sampled | Y | N | Y, (5.5–7mm, The Natural History Museum London Department of Botany: BM000945027) | N | Y | Y | Y |
| 29 | *U. nervosa* | Not sampled | Y | N | Y, (ca. 7.5mm, Herbarium Genavense: G00436785) | Y | N | Y | Y |
| 30 | *U. orientalis* | Not sampled | Y | N | Y, (ca. 7mm, Queensland Herbarium: BRI-AQ0294685) | ？ | Y | Y | Y |
| 31 | *U. ovata* | Not sampled | Y | ? | Y, (ca. 7.5mm, Herbarium Genavense: G00436789) | N | ? | Y | Y |
| 32 | *U. perrottetii* | Not sampled | Y | N | Y, (8–10mm, The Natural History Museum London Department of Botany: BM000945023) | Y | N | N | N |
| 33 | *U. roxburghiana* | Not sampled | N | Y | ？ | Y | N | Y | ？ |
| 34 | *U. schlenckerae* | Not sampled | Y | N | Y, (ca. 8mm, Queensland Herbarium: BRI-AQ0278772) | Y | N | Y | Y |
| 35 | *U. sterrophylla* | Not sampled | Y | N | ？ | ? | N | Y | Y |
| 36 | *U. velutina* | Not sampled | Y | N | ？ | Y | N | Y | Y |
| 37 | *U. domatiifera* | Not sampled | Y | N | Y, (ca. 7mm, Herb. Horti Bot. Nat. Belg.: BR885863) | N | N | Y | Y |
| 38 | *U. talbotii* | Not sampled | Y | N | Y, (20–23mm, The Natural History Museum London Department of Botany: BM000902814) | Y | N | Y | Y |

**Table S4.** Dispersal constraints between different regions for the BioGeoBEARS analysis of *Uncaria* and its close sister taxa. The dispersal probabilities among four regions were specified to three categories for two time slices (20–7 Ma, 7–0 Ma): 1 for dispersal between adjacent areas without barriers, 0.5 for dispersal between areas separated by intermittent barriers, and 0.01 for highly unlikely dispersal events.

|  |  | **Asia** | **Tropical Oceania** | **Tropical Africa** | **Tropical America** |
| --- | --- | --- | --- | --- | --- |
| 7-0 Ma | Asia | 1 | 1 | 1 | 0.01 |
|  | tropical Oceania | 1 | 1 | 0.5 | 0.01 |
|  | tropical Africa | 1 | 0.5 | 1 | 0.01 |
|  | tropical America | 0.01 | 0.01 | 0.01 | 1 |
| 20-7 Ma | Asia | 1 | 1 | 1 | 0.5 |
|  | tropical Oceania | 1 | 1 | 0.5 | 0.01 |
|  | tropical Africa | 1 | 0.5 | 1 | 0.5 |
|  | tropical America | 0.5 | 0.01 | 0.5 | 1 |

**Table S5.** The model selection results under M0 and M1 models from BioGeoBEARS analysis.

|  | **Model** | **LnL** | **numparams** | **d** | **e** | **j** | **AICc** | **AICc_wt** |
| --- | --- | --- | --- | --- | --- | --- | --- | --- |
| M0 | DEC | -27.48 | 2 | 0.012 | 0.0031 | 0 | 59.58 | 0.26 |
|  | DEC+J | -26.34 | 3 | 0.0083 | 1.00E-12 | 0.021 | 60.01 | 0.21 |
|  | **DIVALIKE** | **-27.19** | **2** | **0.013** | **1.00E-12** | **0** | **59.02** | **0.34** |
|  | DIVALIKE+J | -26.56 | 3 | 0.01 | 1.00E-12 | 0.016 | 60.46 | 0.17 |
|  | BAYAREALIKE | -32.73 | 2 | 0.014 | 0.023 | 0 | 70.09 | 0.0013 |
|  | BAYAREALIKE+J | -28.53 | 3 | 0.0072 | 1.00E-07 | 0.03 | 64.4 | 0.023 |
| M1 | DEC | -25.39 | 2.00 | 0.02 | 0.00 | 0.00 | 55.40 | 0.24 |
|  | DEC+J | -24.39 | 3.00 | 0.01 | 1.0e−12 | 0.03 | 56.11 | 0.17 |
|  | **DIVALIKE** | **-24.88** | **2.00** | **0.02** | **1.0e**−**12** | **0.00** | **54.40** | **0.40** |
|  | DIVALIKE+J | -24.42 | 3.00 | 0.02 | 1.0e−12 | 0.02 | 56.17 | 0.17 |
|  | BAYAREALIKE | -30.90 | 2.00 | 0.02 | 0.02 | 0.00 | 66.43 | 0.00 |
|  | BAYAREALIKE+J | -26.63 | 3.00 | 0.01 | 1.0e−07 | 0.04 | 60.60 | 0.02 |

Note: The best fit model is shown in bold.

**Table S7.** The regularization multiplier and feature class combinations for four clades of *Uncaria*.

| **Clade** | **Feature class combinations** | **Regularization multiplier** |
| --- | --- | --- |
| Clade I | LQHP | 0.5 |
| Clade II | LQHP | 1 |
| Clade III | LQ | 0.5 |
| Clade IV | LQHP | 0.5 |

Note: L (Linear), automatic linear; Q, quadratic; H (hinge), fragmentation; P, product in feature class combinations.

**Table S8.** The importance of 27 environmental factors for *Uncaria* from MaxEnt v.3.4.4.

| **Number** | **Variable** | **Percent contribution** | **Permutation importance** |
| --- | --- | --- | --- |
| 1 | bio16 | 56.1 | 7.4 |
| 2 | bio12 | 21.2 | 7.3 |
| 3 | downward surface shortwave radiation | 5.8 | 15.6 |
| 4 | bio17 | 3.8 | 0.8 |
| 5 | bio13 | 2.9 | 8.6 |
| 6 | soil texture classification by USDA | 1.7 | 3.3 |
| 7 | bio14 | 1.7 | 1.4 |
| 8 | bio18 | 1 | 2.3 |
| 9 | bio15 | 0.9 | 3.5 |
| 10 | bio2 | 0.7 | 2 |
| 11 | bio3 | 0.7 | 1 |
| 12 | vapor pressure | 0.5 | 3.8 |
| 13 | bio8 | 0.5 | 6.3 |
| 14 | sand content | 0.4 | 3.2 |
| 15 | bio4 | 0.4 | 4.1 |
| 16 | wind speed | 0.4 | 1.3 |
| 17 | bio7 | 0.3 | 19.5 |
| 18 | bio5 | 0.3 | 0.1 |
| 19 | bio10 | 0.1 | 2.2 |
| 20 | bio19 | 0.1 | 2.2 |
| 21 | bio11 | 0.1 | 0 |
| 22 | soil pH in water | 0.1 | 0.9 |
| 23 | bio9 | 0.1 | 2.1 |
| 24 | bio6 | 0.1 | 0.1 |
| 25 | bio1 | 0.1 | 0 |
| 26 | total nitrogen content | 0 | 0.4 |
| 27 | organic carbon content | 0 | 0.6 |

**Table S9.** Types and abundance of SSR for *Uncaria* and its sister taxa.

| **Number** | **Taxon name** | **A** | **T** | **C** | **G** | **AT** | **TA** | **CT** | **TAT** | **TAA** | **TTA** | **TTC** | **AAG** | **AAT** | **ATT** | **AAAG** | **AAAT** | **AATA** | **AAGT** | **ATAA** | **CAAA** | **GTCT** | **TCAA** | **TTAC** | **TAGG** | **AATTA** | **AGAAT** | **ATCTA** | **ACAAA** | **CTAAA** | **TAAAG** | **TAAAA** | **TTTCT** | **TTTTA** | **AAATAG** |
| --- | --- | --- | --- | --- | --- | --- | --- | --- | --- | --- | --- | --- | --- | --- | --- | --- | --- | --- | --- | --- | --- | --- | --- | --- | --- | --- | --- | --- | --- | --- | --- | --- | --- | --- | --- |
| 1 | *Uncaria homomalla* OQ679828 | 15 | 22 | 1 | 1 | 2 | 2 | 1 | 2 | 1 | 1 | 1 | 0 | 0 | 0 | 1 | 1 | 0 | 0 | 0 | 0 | 1 | 1 | 0 | 0 | 0 | 0 | 0 | 0 | 0 | 0 | 0 | 0 | 0 | 0 |
| 2 | *Uncaria homomalla* PJY26 | 13 | 25 | 1 | 1 | 2 | 2 | 1 | 2 | 1 | 1 | 1 | 0 | 0 | 0 | 1 | 1 | 0 | 1 | 0 | 0 | 1 | 1 | 0 | 0 | 0 | 0 | 0 | 0 | 0 | 0 | 0 | 0 | 0 | 0 |
| 3 | *Uncaria rhynchophylla* PJY12 | 16 | 28 | 1 | 1 | 2 | 2 | 1 | 2 | 1 | 1 | 1 | 0 | 0 | 0 | 1 | 1 | 0 | 1 | 0 | 0 | 1 | 1 | 0 | 0 | 0 | 0 | 0 | 0 | 0 | 0 | 0 | 0 | 0 | 0 |
| 4 | *Uncaria rhynchophylla* MT991006 | 17 | 27 | 1 | 1 | 2 | 2 | 1 | 2 | 1 | 1 | 1 | 0 | 0 | 0 | 1 | 1 | 0 | 1 | 0 | 0 | 1 | 1 | 0 | 0 | 0 | 0 | 0 | 0 | 0 | 0 | 0 | 0 | 0 | 0 |
| 5 | *Uncaria rhynchophylla* OQ679830 | 17 | 28 | 1 | 1 | 2 | 2 | 1 | 2 | 1 | 1 | 1 | 0 | 0 | 0 | 1 | 1 | 0 | 1 | 0 | 0 | 1 | 1 | 0 | 0 | 0 | 0 | 0 | 0 | 0 | 0 | 0 | 0 | 0 | 0 |
| 6 | *Uncaria scandens* ON243637 | 15 | 25 | 1 | 1 | 2 | 2 | 1 | 2 | 1 | 1 | 1 | 0 | 0 | 0 | 1 | 1 | 0 | 1 | 0 | 0 | 1 | 1 | 0 | 0 | 0 | 0 | 0 | 0 | 0 | 0 | 0 | 0 | 0 | 0 |
| 7 | *Uncaria hirsuta* HXH79 | 17 | 26 | 1 | 1 | 2 | 2 | 1 | 1 | 1 | 1 | 1 | 0 | 0 | 0 | 1 | 1 | 0 | 1 | 0 | 0 | 1 | 1 | 0 | 0 | 0 | 0 | 0 | 0 | 0 | 0 | 0 | 0 | 0 | 0 |
| 8 | *Uncaria hirsuta* HXH74 | 17 | 24 | 1 | 1 | 2 | 3 | 1 | 1 | 1 | 1 | 1 | 0 | 0 | 0 | 1 | 1 | 0 | 1 | 0 | 0 | 1 | 1 | 0 | 0 | 0 | 0 | 0 | 0 | 0 | 0 | 0 | 0 | 0 | 0 |
| 9 | *Uncaria hirsuta* OQ679827 | 17 | 26 | 1 | 1 | 2 | 2 | 1 | 1 | 1 | 1 | 1 | 0 | 0 | 0 | 1 | 1 | 0 | 1 | 0 | 0 | 1 | 1 | 0 | 0 | 0 | 0 | 0 | 0 | 0 | 0 | 0 | 0 | 0 | 0 |
| 10 | *Uncaria sinensis* PJY18 | 18 | 34 | 0 | 0 | 2 | 2 | 1 | 2 | 0 | 1 | 1 | 0 | 0 | 0 | 0 | 1 | 0 | 1 | 0 | 0 | 1 | 1 | 0 | 0 | 0 | 0 | 0 | 0 | 0 | 0 | 0 | 0 | 0 | 0 |
| 11 | *Uncaria sinensis* OQ679832 | 18 | 34 | 0 | 0 | 2 | 2 | 1 | 2 | 0 | 1 | 1 | 0 | 0 | 0 | 0 | 1 | 0 | 1 | 0 | 0 | 1 | 1 | 0 | 0 | 0 | 0 | 0 | 0 | 0 | 0 | 0 | 0 | 0 | 0 |
| 12 | *Uncaria rhynchophylloides* PJY17 | 14 | 27 | 1 | 1 | 2 | 2 | 1 | 2 | 1 | 1 | 1 | 0 | 0 | 0 | 0 | 1 | 0 | 1 | 0 | 0 | 1 | 1 | 0 | 0 | 0 | 0 | 0 | 0 | 0 | 0 | 0 | 0 | 0 | 0 |
| 13 | *Uncaria rhynchophylloides* OQ679831 | 15 | 29 | 1 | 0 | 2 | 2 | 1 | 2 | 1 | 1 | 1 | 0 | 0 | 0 | 0 | 1 | 0 | 1 | 0 | 0 | 1 | 1 | 0 | 0 | 0 | 0 | 0 | 0 | 0 | 0 | 0 | 0 | 0 | 0 |
| 14 | *Uncaria lancifolia* PJY05 | 10 | 24 | 0 | 0 | 2 | 3 | 0 | 2 | 1 | 0 | 1 | 0 | 0 | 0 | 0 | 1 | 0 | 1 | 0 | 0 | 1 | 1 | 0 | 0 | 1 | 0 | 0 | 0 | 0 | 1 | 0 | 0 | 0 | 0 |
| 15 | *Uncaria lancifolia* PJY15 | 11 | 22 | 0 | 0 | 2 | 2 | 1 | 2 | 1 | 0 | 1 | 0 | 0 | 0 | 0 | 1 | 0 | 1 | 0 | 0 | 1 | 1 | 0 | 0 | 0 | 0 | 1 | 0 | 0 | 0 | 0 | 0 | 0 | 0 |
| 16 | *Uncaria lancifolia* OQ679829 | 12 | 22 | 0 | 0 | 2 | 2 | 1 | 2 | 1 | 0 | 1 | 0 | 0 | 0 | 0 | 1 | 0 | 1 | 0 | 0 | 1 | 1 | 0 | 0 | 0 | 0 | 1 | 0 | 0 | 0 | 0 | 0 | 0 | 0 |
| 17 | *Uncaria macrophylla* ON243636 | 9 | 25 | 0 | 0 | 3 | 2 | 1 | 2 | 1 | 0 | 1 | 0 | 0 | 0 | 0 | 1 | 0 | 1 | 0 | 0 | 1 | 1 | 0 | 0 | 0 | 0 | 0 | 0 | 0 | 1 | 0 | 0 | 0 | 0 |
| 18 | *Uncaria macrophylla* MZ869757 | 10 | 23 | 0 | 0 | 3 | 2 | 1 | 2 | 1 | 0 | 1 | 0 | 0 | 0 | 0 | 1 | 0 | 1 | 0 | 0 | 1 | 1 | 0 | 0 | 0 | 0 | 0 | 0 | 0 | 1 | 0 | 0 | 0 | 0 |
| 19 | *Uncaria macrophylla* PJY13 | 10 | 23 | 0 | 0 | 3 | 2 | 1 | 2 | 1 | 0 | 1 | 0 | 0 | 0 | 0 | 1 | 0 | 1 | 0 | 0 | 1 | 1 | 0 | 0 | 0 | 0 | 0 | 0 | 0 | 1 | 0 | 0 | 0 | 0 |
| 20 | *Uncaria lanosa* f. *philippinensis* PJY04 | 13 | 25 | 0 | 0 | 2 | 3 | 1 | 2 | 1 | 0 | 1 | 0 | 0 | 0 | 0 | 1 | 0 | 1 | 0 | 0 | 1 | 0 | 0 | 0 | 0 | 0 | 0 | 0 | 0 | 1 | 0 | 0 | 0 | 0 |
| 21 | *Uncaria lanosa* var. *appendiculat* HXH115 | 11 | 26 | 1 | 0 | 2 | 2 | 1 | 2 | 1 | 0 | 1 | 0 | 0 | 0 | 0 | 1 | 0 | 1 | 0 | 0 | 1 | 0 | 0 | 0 | 0 | 0 | 0 | 0 | 0 | 1 | 0 | 0 | 0 | 0 |
| 22 | *Uncaria callophylla* HXH118 | 10 | 28 | 1 | 0 | 2 | 3 | 1 | 2 | 1 | 0 | 1 | 0 | 0 | 0 | 0 | 0 | 0 | 1 | 0 | 0 | 1 | 1 | 1 | 0 | 0 | 0 | 0 | 0 | 0 | 1 | 0 | 1 | 0 | 1 |
| 23 | *Uncaria sessilifructus* ON243635 | 15 | 23 | 1 | 0 | 2 | 1 | 1 | 2 | 1 | 0 | 1 | 0 | 0 | 0 | 0 | 1 | 1 | 1 | 1 | 0 | 1 | 1 | 0 | 0 | 0 | 0 | 0 | 0 | 0 | 1 | 1 | 0 | 1 | 0 |
| 24 | *Uncaria laevigata* PJY14 | 12 | 25 | 1 | 0 | 2 | 1 | 1 | 2 | 1 | 0 | 1 | 0 | 0 | 0 | 0 | 1 | 1 | 1 | 0 | 0 | 1 | 1 | 0 | 0 | 0 | 0 | 0 | 0 | 0 | 1 | 0 | 0 | 0 | 0 |
| 25 | *Uncaria guianensis* OP794339 | 10 | 21 | 0 | 1 | 2 | 1 | 1 | 2 | 1 | 0 | 1 | 0 | 0 | 0 | 0 | 1 | 0 | 0 | 0 | 0 | 1 | 1 | 0 | 0 | 0 | 0 | 0 | 0 | 0 | 0 | 0 | 0 | 0 | 0 |
| 26 | *Uncaria tomentosa* OP794340 | 8 | 23 | 1 | 0 | 2 | 3 | 1 | 0 | 1 | 0 | 1 | 1 | 1 | 0 | 0 | 0 | 0 | 0 | 0 | 0 | 1 | 1 | 0 | 0 | 0 | 0 | 0 | 0 | 0 | 0 | 0 | 0 | 0 | 0 |
| 27 | *Uncaria angolensis* PJY10 | 11 | 16 | 0 | 0 | 2 | 2 | 1 | 3 | 1 | 0 | 1 | 0 | 0 | 0 | 0 | 0 | 0 | 0 | 0 | 1 | 1 | 1 | 0 | 0 | 0 | 0 | 0 | 0 | 0 | 0 | 0 | 0 | 0 | 0 |
| 28 | *Uncaria africana* PJY19 | 12 | 17 | 0 | 0 | 2 | 2 | 1 | 2 | 1 | 0 | 1 | 0 | 0 | 0 | 0 | 0 | 0 | 0 | 0 | 0 | 1 | 1 | 0 | 0 | 0 | 0 | 0 | 0 | 0 | 0 | 0 | 0 | 0 | 0 |
| 29 | *Uncaria donisii* PJY20 | 8 | 19 | 0 | 0 | 2 | 2 | 1 | 2 | 1 | 0 | 1 | 0 | 0 | 1 | 0 | 0 | 0 | 0 | 0 | 0 | 1 | 1 | 0 | 0 | 0 | 0 | 0 | 1 | 0 | 0 | 0 | 0 | 0 | 0 |
| 30 | *Neolamarckia cadamba* MG572117 | 14 | 21 | 1 | 1 | 2 | 1 | 1 | 1 | 1 | 0 | 0 | 0 | 0 | 0 | 0 | 0 | 0 | 1 | 0 | 0 | 1 | 1 | 0 | 0 | 0 | 1 | 0 | 0 | 0 | 0 | 0 | 0 | 0 | 0 |
| 31 | *Neolamarckia cadamba* HXH82 | 14 | 21 | 1 | 1 | 2 | 1 | 1 | 1 | 1 | 0 | 0 | 0 | 0 | 0 | 0 | 0 | 0 | 1 | 0 | 0 | 1 | 1 | 0 | 0 | 0 | 1 | 0 | 0 | 0 | 0 | 0 | 0 | 0 | 0 |
| 32 | *Neolamarckia macrophylla* MN877388 | 11 | 15 | 1 | 1 | 2 | 1 | 1 | 3 | 0 | 0 | 1 | 0 | 0 | 0 | 0 | 1 | 0 | 1 | 0 | 0 | 1 | 1 | 0 | 0 | 0 | 1 | 0 | 0 | 0 | 0 | 0 | 0 | 0 | 0 |
| 33 | *Nauclea latifolia* PJY23 | 10 | 23 | 1 | 0 | 3 | 1 | 1 | 2 | 0 | 0 | 1 | 0 | 0 | 0 | 0 | 1 | 0 | 1 | 0 | 0 | 1 | 1 | 0 | 0 | 0 | 1 | 0 | 0 | 1 | 0 | 0 | 0 | 0 | 0 |
| 34 | *Nauclea orientalis* HXH122 | 14 | 22 | 1 | 0 | 1 | 1 | 1 | 1 | 1 | 0 | 1 | 0 | 0 | 0 | 0 | 1 | 0 | 1 | 0 | 0 | 1 | 1 | 0 | 1 | 0 | 1 | 0 | 0 | 0 | 0 | 0 | 0 | 0 | 0 |

**Table S10.** Posterior age distributions for key nodes of *Uncaria* under different calibration protocols. Node numbers correspond to those in Fig. 3.

| **Node** | **Normal prior** | |  | **Exponential prior** | |  | **Lognormal prior** | |
| --- | --- | --- | --- | --- | --- | --- | --- | --- |
|  | **Mean (Ma)** | **95% HPD (Ma)** |  | **Mean (Ma)** | **95% HPD (Ma)** |  | **Mean (Ma)** | **95% HPD (Ma)** |
| 1: *Uncaria* stem | 19.03 | 24.70–13.57 |  | 19.79 | 26.05–13.66 |  | 20.65 | 27.25–14.34 |
| 2: *Uncaria* crown | 17.79 | 23.31–12.65 |  | 18.50 | 24.40–12.61 |  | 19.30 | 25.65–13.34 |
| 3: *Uncaria* Clade I crown | 10.59 | 14.62–6.95 |  | 10.98 | 15.49–6.77 |  | 11.42 | 16.06–7.19 |
| 4: *Uncaria* Clade II crown | 8.36 | 12.49–4.40 |  | 8.65 | 13.31–4.27 |  | 8.94 | 13.33–4.59 |
| 5: *Uncaria* Clade III crown | 5.15 | 9.74–1.41 |  | 5.29 | 10.14–1.31 |  | 5.54 | 10.71–1.44 |
| 6: *Uncaria* Clade IV crown | 11.56 | 16.72–6.78 |  | 12.05 | 17.77–6.71 |  | 12.49 | 18.67–6.96 |

**Table S11.** Summary counts of 100 BSMs from BioGeoBEARS results.

|  | **founder** | **a** | **d** | **e** | **subset** | **vicariance** | **sympatry** | **ALL_disp** | **ana_disp** | **all_ana** | **all_clado** | **total_events** |
| --- | --- | --- | --- | --- | --- | --- | --- | --- | --- | --- | --- | --- |
| means | 0 | 0 | 7.18 | 0 | 0 | 3.63 | 17.37 | 7.18 | 7.18 | 7.18 | 21 | 28.18 |
| stdevs | 0 | 0 | 0.46 | 0 | 0 | 0.68 | 0.68 | 0.46 | 0.46 | 0.46 | 0 | 0.46 |
| sums | 0 | 0 | 718 | 0 | 0 | 363 | 1737 | 718 | 718 | 718 | 2100 | 2818 |

Note: A, Asia; B, Oceania; C, Africa; D, America. ALL_disp, all dispersal (mean of all observed anagenetic 'a', 'd' dispersals, plus cladogenetic founder/jump dispersal); ana_disp, Anagenetic dispersal (mean of all observed anagenetic 'a' or 'd' dispersals); all_ana, all Anagenetic (mean of all observed 'a', 'd' and 'e'); all_clado, all Cladogenetic (mean of all sympatries, plus founder, and vicariance).

**Table S12.** Mean number of dispersal events per source and sink after 100 BSMs from BioGeoBEARS results for *Uncaria* and the closest sister taxa.

|  | **Asia** | **tropical Oceania** | **tropical Africa** | **tropical America** |
| --- | --- | --- | --- | --- |
| Asia | 0 | 3.94 | 1.26 | 0.25 |
| tropical Oceania | 0.17 | 0 | 0.02 | 0 |
| tropical Africa | 0.36 | 0.02 | 0 | 0.59 |
| tropical America | 0.16 | 0 | 0.41 | 0 |
